# New integrative methodology for the excavation and study of mass graves and partially commingled remains

**DOI:** 10.1371/journal.pone.0353420

**Published:** 2026-07-17

**Authors:** Marcos De Andrés Montero, Luca Kis, Olga Spekker, Viktor Vig, Réka Kocsmár, Zsófia Simon, Béla Simon, Réka Neményi, Gábor Bertók, Balázs Tihanyi, György Pálfi

**Affiliations:** 1 Department of Biological Anthropology, Institute of Biology, University of Szeged, Szeged, Hungary; 2 Department of Archaeology, Janus Pannonius Museum, Pécs, Hungary; 3 Town Museum Library and Archive of Kőszeg, Kőszeg, Hungary; 4 Department of Archaeology, University of Szeged, Szeged, Hungary; Institute for Anthropological Research, CROATIA

## Abstract

The resolution of commingled human skeletal remains is a phenomenon increasingly encountered by archaeologists and anthropologists. Its difficulty is due to the complexity of the task itself, but also to the limited methodological resources available for researchers. Parallelly, its importance derives from the fact that individualized skeletons are a prerequisite for any anthropological analysis that aims to provide results. In this paper, we put forward a new methodological approach that aims to integrate different tools and techniques already available. The basis of this methodology is the analysis of the spatial relationships of the human skeletal remains. To facilitate its use and maximize its effectiveness, we have developed a set of specific tools and protocols that include a list of information to be directly recorded in Geographical Information System (GIS) software, as well as a new coding system for skeletal remains showing different levels of completeness. Results show that the application of such a methodology helped in the resolution of a large, commingled assemblage exhumed from a 16^th^-century-CE burial located in southwestern Hungary. The original number of complete skeletons (those individualized in their entirety during the fieldwork) was substantially increased, and for several other skeletal elements, potential matches were found during the laboratory work. We found this methodology to be a good filter prior to the utilization of the more standardized techniques such as osteometric sorting, articulation, visual pair-matching or process of elimination, as it would increase their effectiveness. Additionally, the novel coding system has proven to be useful in inventorying and storing human skeletal remains. This project shows that the addition of GIS technology and spatial analysis does increase the success rate in the reassociation of commingled human skeletal remains.

## Introduction

The excavation, exhumation, and examination of graves containing the remains of multiple individuals represent one of the most challenging tasks faced by professionals in forensic anthropology, forensic archaeology, or osteoarchaeology. Such graves have been documented as early as the Neolithic period [1,2]. Despite their continuous discovery over recent decades [3–8], and the growing importance of these contexts for the investigation of recent conflicts and human rights abuses [9–14], two significant shortcomings persist in the theoretical approach to their study.

First, despite several attempts in recent years aiming to clarify and unify the terminology and classification of graves containing more than one individual [15–18], no system has gained general acceptance across the disciplines concerned. Inconsistent terminologies are due to differences in vocabulary in the different national traditions, as well as distinct theoretical approaches. While the French tradition has developed a more stable and accurate terminology, with concepts built maintaining a clear distinction between purely descriptive observation and interpretation; the English-speaking world has collected a variety of terms, sometimes overlapping each other, derived from a number of notions produced based on descriptions, interpretations or a mixture of both [16,19]. However, scholars from different traditions have emphasized the need to distinguish between different types of plural interments [15,16]. Following Boulestin & Courtaud [16], the more general term “plural burial” is to be utilised when it is not possible to establish the number of depositional events. Within this framework, a “multiple burial” refers to a context where all bodies were interred in a single event, while a “collective burial” describes a burial containing bodies deposited over more than one event. A mass grave is defined as a subtype of multiple burial, usually associated with contemporary episodes of mass violence [16]. Kay & Koncz [15] based their distinction between multiple burial and mass grave on the functionality of the site, considering that mass graves are purely functional sites where bodies are disposed following patterns that would be non-normative for the social group that created the site, this could include episodes of violence, but also epidemics, famines, and other events that would require a fast massive disposition of a large number of cadavers. On the other hand, multiple burials would follow social practices alike to those of a single burial [15]. Despite these distinctions, authors tend to use the term mass grave in both archaeological and forensic contexts as a sort of umbrella term [1,20–23] often interchangeably with “mass burial”, or “multiple burial”, but with no solid analysis nor reflection on the adequacy of this denomination for each case, resulting in the obfuscation of the term.

In recent decades the concept of mass grave has been widely discussed on its own, with various approaches and definitions proposed. However, there remains little consensus on the criteria that define such a burial [24]. Skinner [25] proposed that a mass grave must contain “at least half a dozen individuals”. Later, Jesse & Skinner [26] offered a new definition, suggesting that “a mass grave is any location containing two or more associated bodies, indiscriminately or deliberately placed, of victims who have died as a result of extra-judicial, summary or arbitrary executions, not including those individuals who have died as a result of armed confrontations or known major catastrophes”. They further developed a classification system for mass graves and their associated sites [26]. Nevertheless, as noted by Haglund & colleagues [27], a mass grave can also be the result of a high number of casualties after natural disasters. It is important to note that these definitions were developed to serve legal processes related to recent human rights violations, in which the authors were involved as expert witnesses; and therefore, their definitions are based on attributes such as the number of corpses, the context of their demise, or the relationship between the bodies interred in these type of burials [28]. Even if they include archaeological features, their main objective has been compliance with international legal standards [26]. Consequently, these definitions may be less applicable when dealing with burials that are several centuries old.

Second, in addition to conceptual inconsistencies, there is also a lack of generally applicable methodologies for the exhumation and examination of skeletal remains recovered from mass graves [29]. The variability in the physical features of each mass grave, combined with the diversity of post-depositional processes affecting them, has led teams across the world to develop case-specific methodologies [27,29,30]. While practical, this approach greatly limits both the possibilities for comparative studies on grave typology, depositional composition, or the taphonomic damage observed, as well as the applicability of those case-specific methods.

Distinct variables can potentially complicate the excavation and exhumation of remains interred in any burial, and specially those containing multiple individuals [31]. The localization of the burial can increase the difficulty of the process if the access to it is challenging; in addition, the climate is another factor to be considered when approaching the excavation of a site containing large numbers of remains as it can have a considerable impact in the amount of time that is available for the completion of the work. The structure of the grave, the dispersion of the remains, and the damage already suffered by the site are also features that will determine the difficulty of each case. Choosing the right excavation methodology can influence the result of the work as well, and it has been demonstrated that the amount of skeletal material that loses its connection varies depending on the chosen methodology [32].

A characteristic frequently encountered in plural burials of any kind is the presence of commingling – the mixing of skeletal elements from different individuals into a single assemblage [33]. Such mixing may occur at any stage of the post-mortem interval through a wide range of natural or anthropogenic processes [34]. As a result, small-scale or large-scale assemblages can form, and a number of four individuals has been proposed as the threshold [35]. The size of the assemblage has direct implications for both the magnitude of the challenge and the effectiveness of the available methodological tools [33–35].

Addressing this problem is essential for the successful completion of any anthropological analysis. As noted by several authors [36–38], developing biological profiles or determining the cause and manner of death – particularly in cases of forensic interest – is not possible in commingled assemblages unless the skeletal remains are first segregated and reassociated into individuals.

Some published protocols (e.g., [39,40]) and compilations of different case studies [41,42] provide valuable guidelines for the excavation and exhumation of mass graves, including those containing commingled remains. These protocols emphasize the importance of exhaustive documentation of the grave, the remains, and the entire recovery process, which is indeed the most optimal standard. However, they rarely address how such exhaustive documentation can realistically be achieved when time and financial resources are both limited. In practice, researchers often face a dilemma between two competing priorities: ensuring the best preservation of the remains, which generally requires minimizing the time they are exposed, or maximizing the precision of the field documentation, which is inherently time-intensive. While this tension is less problematic in small-scale plural burials – where detailed documentation can be completed within a reasonable timeframe –, larger assemblages, particularly those involving commingling, exponentially increase the complexity of procedures. Under such circumstances, it becomes impossible to simultaneously achieve the goals of preservation and comprehensive documentation. This problem underscores the necessity of developing methodologies explicitly designed to maximalize the quantity and quality of recoverable information with the shortest possible timeframe.

A central issue, both in the field and in the laboratory, is therefore resolving commingling and reassociating the dispersed skeletal elements into individual skeletons. Several methodologies and tools have been applied to various cases involving plural burials and commingled remains both in forensic and archaeological contexts, and while they have proved useful in some cases, they also have important limitations that become especially notorious when the case at hand consists of large-size assemblages [43].

The documentation of plural burials is most commonly conducted following standard archaeological practices, with total stations remaining the predominant method for recording the spatial position of elements during fieldwork. However, several studies have reported the use of Geographical Information System (GIS) software for the “storage, analysis, and display of spatial data” [44] as a complementary tool in the documentation process, integrating and combining GIS with more standardized methods such as total station or photography [29]. In recent years, GIS has been increasingly used in archaeological and anthropological research for a variety of purposes, including the localization of recent mass graves resulting from contemporary conflicts or episodes of violence [45,46]; the calculation of the minimum number of individuals (MNI) in highly fragmented and commingled assemblages [47]; the recording of perimortem trauma [48]; and the reassociation of commingled or mixed skeletal remains through spatial analytical approaches [49,50]. In addition, GIS was successfully used to test the validity of the hypothesis that bones forming an articulation and found in close proximity are likely to belong to the same individual [49,50]. Nevertheless, these studies emphasized the need to integrate additional analytical techniques further strengthening the reliability of reassociation results.

A variety of techniques are available for resolving commingled assemblages and are typically applied during the laboratory phase, these include: 1) visual pair-matching, in which homologous bones are associated based on morphological similarity [51–53]; 2) articulation, which conjoins bones that adequately fit together at articular surfaces [51,52]; 3) process of elimination, applied after visual pair-matching and articulation, which assigns the remaining elements to the remaining individuals by exclusion [51,52]; 4) osteometric sorting, which uses statistical models to compare measurements describing size and shape of elements, segregating those incompatible with a given individual [36–38,51,54]; 5) taphonomy analysis, which considers post-mortem alterations as a basis for separating elements [40]; 6) age, sex, and pathological features, which in some contexts serve as individualizing characteristics that guide reassociation [40]; and 7) DNA and other molecular analyses, which remain among the most reliable methods for reunifying mixed skeletal remains in forensic context [40].

While these techniques are well-established and have demonstrated effectiveness, their reliability can be significantly reduced by the specific characteristics of each burial, such as the size of the assemblage, requiring case-by-case adaptation. At present, there is no comprehensive protocol that addresses these challenges in a systematic manner. Consequently, anthropologists with little or no prior experience of such contexts may find themselves without clear methodological guidance when confronted with unexpectedly commingled remains.

In recent years, our team at the Department of Biological Anthropology, University of Szeged (Szeged, Hungary) has been involved in projects focused on the excavation and analysis of large-scale, partially commingled medieval graves – identified as mass graves according to the criteria outlined by Kay & Koncz [15]. Characterized by hundreds of skeletons, disorderedly thrown into a single grave pit and compacted into a unified skeletal assemblage, these cases present substantial methodological challenges. At the same time, they offer a valuable opportunity to address several of the issues outlined above.

In this paper, we present the methodological approaches and workflow developed by our team to facilitate more effective field and laboratory practices for the documentation, inventory, and reassociation of large-scale, partially commingled skeletal assemblages.

## Materials and methods

### Material

**Fig 1:**
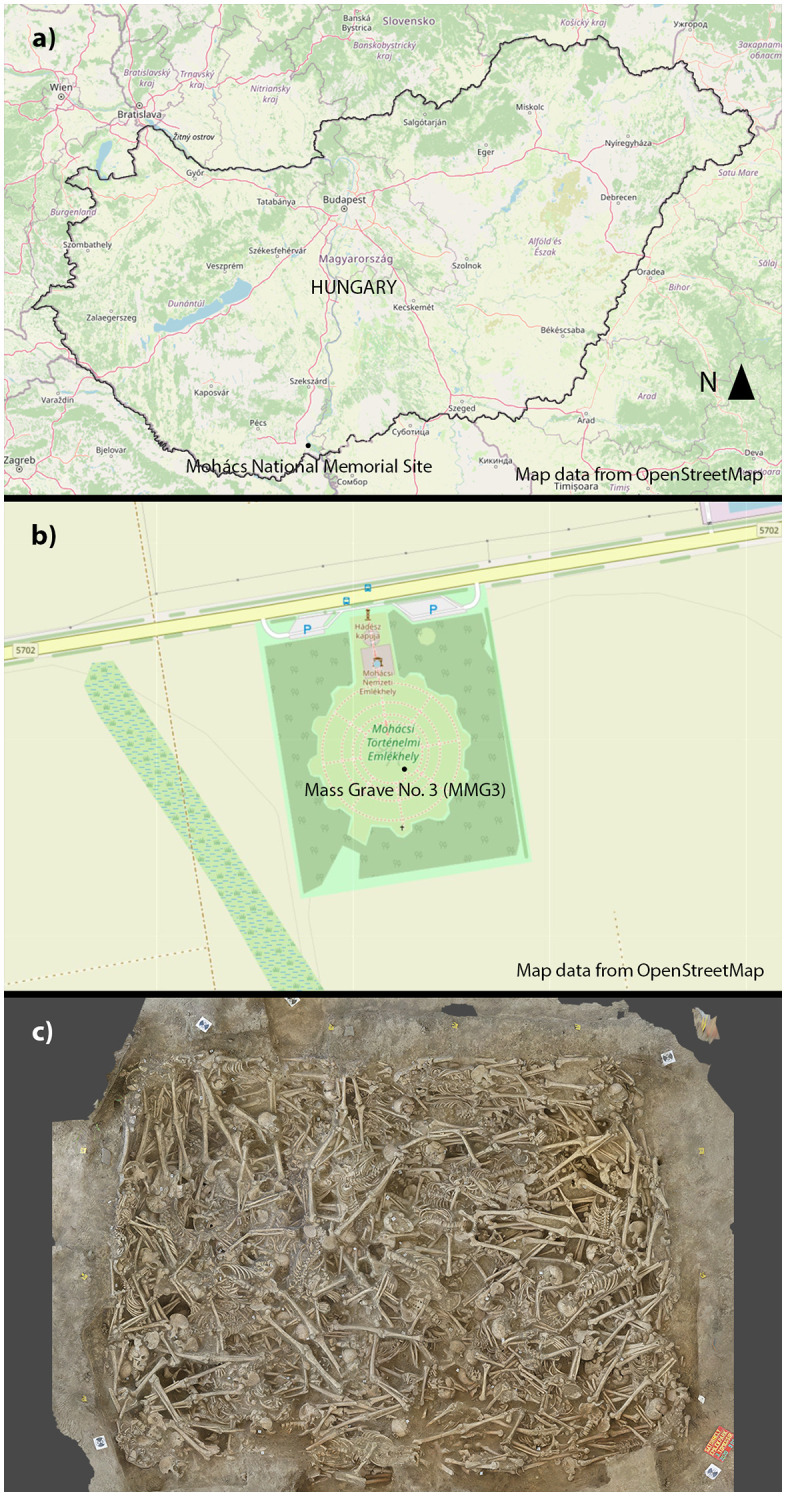
a) Map of Hungary and location of the Mohács National Memorial Site (image obtained from OpenStreetMap and OpenStreetMap Foundation, which is made available under the Open Database License (https://www.openstreetmap.org/copyright)); b) The location of Mass Grave No. 3 within the Mohács National Memorial Site (image obtained from OpenStreetMap and OpenStreetMap Foundation, which is made available under the Open Database License (https://www.openstreetmap.org/copyright)); and c) Orthophoto of Mass Grave No. 3 of the Mohács National Memorial Site (source of the orthophoto: Photo Collection and Archaeological Database of the Janus Pannonius Museum, Pécs, Hungary).

This study is focusing on the examination of human remains exhumed from mass graves at the Mohács National Memorial Site, particularly Mass Grave No. 3 (MMG3), located east of the present-day village of Sátorhely (Baranya County, southwestern Hungary) (Fig 1). These individuals were interred following the Battle of Mohács, which took place on the 29 August 1526 CE. This battle is considered a pivotal event in Hungarian history, marking the beginning of the collapse of the kingdom’s resistance to the ongoing Ottoman conquest attempts that had persisted for nearly two centuries [55–57].

According to historical sources, the Hungarian army opposed a more than 70,000-soldier Ottoman army, and comprised only about 26,000 soldiers [55–57], most of whom perished either in combat or during executions in the following days. Consequently, a substantial number of human remains are expected in the surrounding area. To date, five mass graves have been documented in the vicinity [58]. The first discovery of human bones occurred in the 1950s during the clandestine construction of military facilities. Since 1960, several excavations have taken place. In 1975–1976, during the development of the Mohács National Memorial Site, several hitherto unknown mass graves – including MMG3 – were identified. However, none of these excavations were aimed at removing the human remains from the graves but only exposing the upper surface and, occasionally, some sides of the bone assemblages. The exposed surfaces were documented with the then available techniques of the time, including manual mapping, descriptions, and black-and-white photographs [58]. The excavations of MMG3 conducted between 2020 and 2022 were the first to aim for the complete recovery of the remains [58]. Although the mechanized earthworks disturbed one side of the original pit in 1975, the preserved concentration of skeletal remains formed an irregular rectangle measuring approximately 4 m on the northern side, 4.8 m on the southern side, 3.25 m in width, and 1.4 m in depth, with a total surface area of about 15 m^2^ (Fig 1c) [58]. Preliminary MNI determines that the pit contained the partially commingled remains of roughly 320 individuals [59], deposited without apparent order [58]. Since the bodies had been thrown into the grave pit in a disordered manner, in many cases, various anatomical regions of the same individual were deposited in different layers of the mass grave. Consequently, it was common for some skeletal parts to be uncovered early during the excavation, while others were revealed and recovered only in later stages, making the inventory and reassociation of the skeletons and bone elements particularly challenging [58–60]. Preservation ranged from almost complete, well-preserved skeletons to very incomplete and poorly preserved remains (following the criteria described in [61]), with an overall condition assessed as poor.

Excavation and exhumation were documented systematically according to internationally recognized forensic archaeological protocols [39], adapted to the specific time and budget constraints of the project. Documentation included written descriptions, drawings, photographs, and 3D modelling using photographs of the various surfaces cleaned during the excavation work. Georeferenced orthophotos based on the photographs and 3D models were created during the three excavation seasons and were continuously uploaded to the open-source geographic information system QGIS 3.20.2, creating a chronologically ordered visual record of the excavation process [58]. During fieldwork, individual bone elements shown in the orthophotos were digitally outlined in QGIS by creating polygons in new vector layers, ensuring that the position of thousands of skeletal elements was fixed in a two-dimensional spatial framework. Each outlined element was assigned an inventory number (starting from 1 and added to every set of elements exhumed at once) and an identification serial number based on three categories: 1) individualized skeletons (IDs 1–999), 2) articulated major elements such as upper or lower limbs (IDs 1,000–9,999), and 3) isolated small bones, such as rib fragments or hand bones, recovered without anatomical context (10,000 and above). For each element, the extraction date and potential associations with adjacent remains were recorded in the project database [60]. Additional metadata – such as bone name and laterality – were also part of the recording protocol; however, time constraints in the field and the progressive deterioration of bone preservation limited the completeness of this information.

### Ethics statement

The human skeletons evaluated in the described study are housed in the Department of Biological Anthropology, University of Szeged, in Szeged, Hungary. Access to the specimens was granted by the Department of Biological Anthropology, University of Szeged (Közép fasor 52, H-6726 Szeged, Hungary).

No permits were required for the described study, which complied with all relevant regulations. The research has been conducted in an ethically responsible manner – all bone remains have been examined with dignity and respect.

### Methods

**Fig 2:**
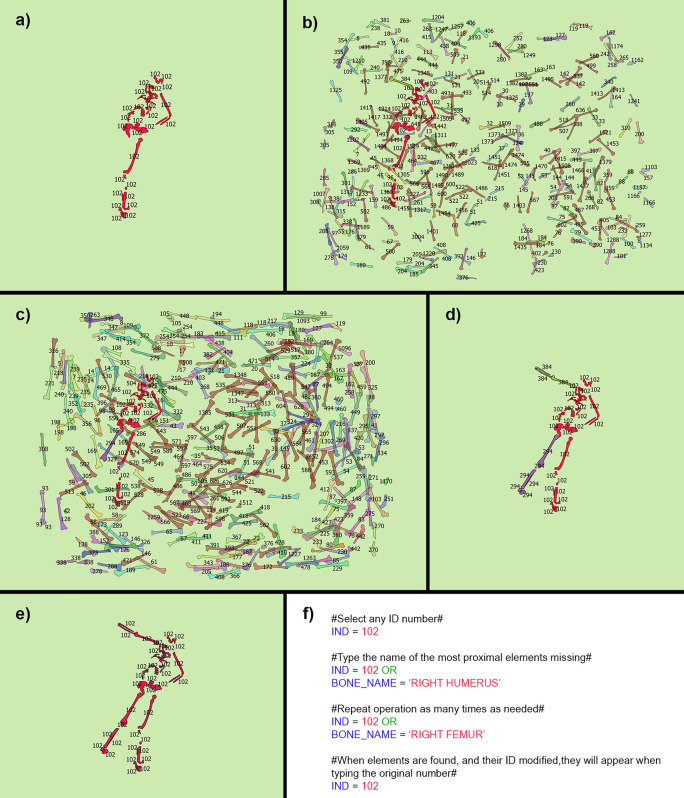
Original step-by-step reassociation workflow in QGIS 3.20.2 where a) shows a single case (102) missing both upper and lower right limbs; b) the same case (102) and all registered right humeri; c) 102 and all registered right femora; d) isolation of 102 and potential matches for this incomplete skeleton, in this case, unit 384 (right humerus) and unit 294 (right femur); e) visualization of 102 after all elements have been assigned to that individual; and f) step-by-step reassociation as it would appear in QGIS 3.20.2 filter tool.

Using the preexisting information (inventory and serial numbers, exhumation dates, and potential matches), the initial attempts at reassociating the commingled elements yielded successful matching in only a limited number of cases within a given time frame. Specifically, a few hundred elements were processed over the course of three months, as the analysis required the labor-intensive, one-by-one comparison of all registered elements with the corresponding missing bones (Fig 2) [59]. In light of these preliminary results, it became evident that a more efficient and less time-consuming method was necessary to process tens of thousands of non-individualized elements. As part of broader research initiative, we began developing a workflow incorporating new tools, approaches, and methodologies. As part of this system, we developed an inventory recording protocol – applicable both in the field and the laboratory – designed to facilitate the more efficient segregation of elements. The protocol consists of the following steps.

### Information per bone element

**Fig 3:**
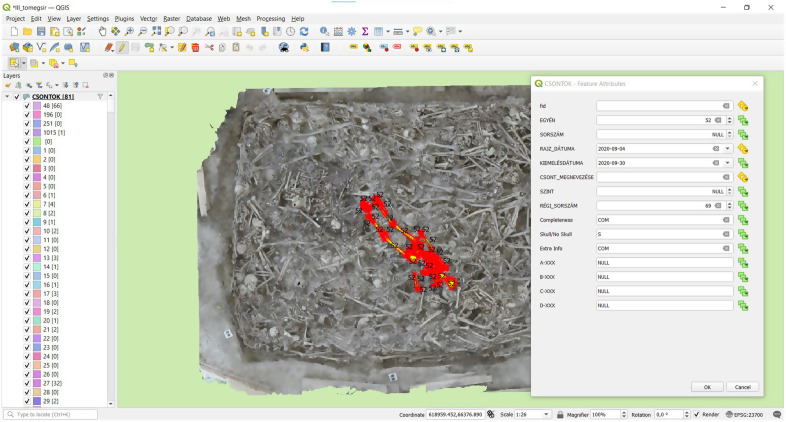
Data recorded in QGIS 3.20.2 for case 52.

Each bone element must contain sufficient information to enable its segregation from the general assemblage as well as its later reassociation with adjacent or related skeletal units. Previous studies have demonstrated that recording features such as inventory number, individual/identification number, laterality, size, robusticity, age, sex, and pathological conditions are particularly relevant when working with commingled skeletal material [39,40,49]. Based on these recommendations, seven key features were selected for the description of bone elements in our workflow: 1) serial number, 2) bone type and laterality, 3) date of removal, 4) possible connections with other elements, 5) code, 6) traceability, and 7) miscellaneous information (Table 1). It should be noted that, in the specific case of MMG3, characteristics such as skeletal sex and age were not included as independent features (Fig 3). Preliminary analyses consistently suggested a certain level of homogeneity regarding both sex and age among the deceased. These aspects, together with any additional relevant details (e.g., particular pathological changes, taphonomic alterations, or exceptional morphological features), were instead subsumed under the seventh category, miscellaneous information.

**Table 1:** Data recorded in QGIS for each set of bones.

Feature	Description	Proposed format
Serial number	*A sequential numbering system starting with 1 and increasing consecutively. The same number was applied to all bone elements within a collected unit, regardless of their type, size or provenience*	00001-99999
Bone type and laterality	*The anatomical identification of the bone element and the side of the body it belongs to. This can be the bone’s full name, or any unique abbreviation for each element*	e.g., “Right Humerus” or “Left Tibia”
Date of removal	*The calendar date when the bone element was recovered from the grave*	format: dd-mm-yyyy e.g., “05-10-2022”
Possible connection with other elements	*If it is observed that a given element may be associated with another, this relationship should be recorded without altering the serial numbering system*	e.g., 250*→*322
Code	*An alphanumeric identifier used to classify skeletal units according to their level of completeness*	COM.S.A12.B2-352
Traceability	*In case a change in the serial number occurs, due to reassociation or segregation, this field allows to register the previous serial number so that changes can be traced. This would allow for mistakes detection and correction*	e.g., 148
Miscellaneous information	*Any additional feature or characteristic observed on an element that may assist in its segregation, such as pathological conditions, perimortem injuries, biological sex, estimated age-at-death, or other individualizing traits*	e.g., “osteomyelitis”, “cut mark”, or “sub-adult”

### Code for the classification of skeletal elements

**Fig 4:**
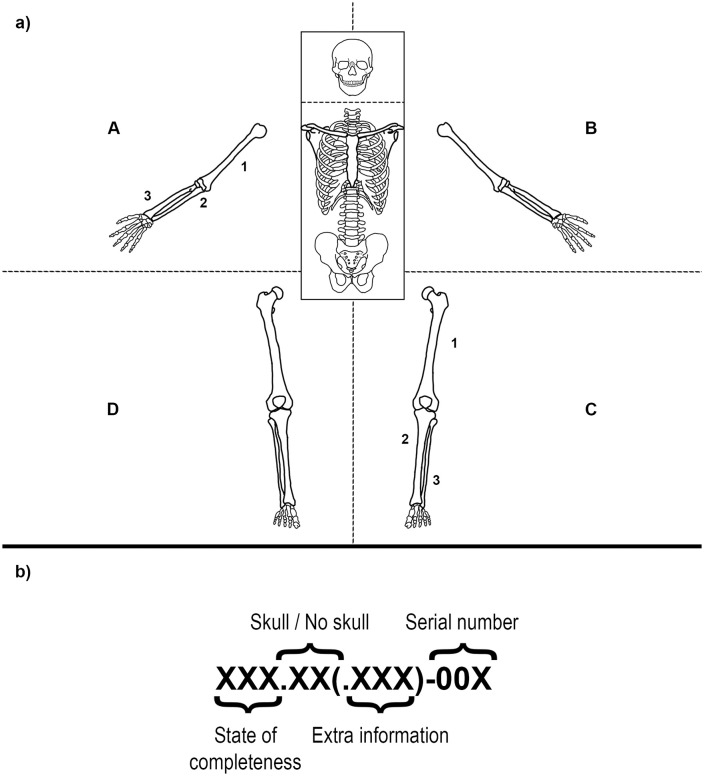
a) Diagram illustrating the separation and coding of limbs and specific bone elements used in the proposed coding system. Letters designate the limbs as follows: A = upper right limb, B = upper left limb, C = lower left limb, and D = lower right limb. Numbers indicate individual bones within each limb: 1 = most proximal element (humerus or femur); 2 = most distal medial bone (ulna or tibia); and 3 = most distal lateral bone (radius or fibula). b) Structure of the proposed coding system. (Diagram created by Marcos De Andrés.).

Including the previously described information for each bone element allows for the identification of potentially related elements. However, one-by-one comparisons of all registered elements with the corresponding missing bones, especially in larger series consisting skeletons with various levels of completeness and preservation, as illustrated in Fig 2, remained challenging. To address this, a new alphanumeric coding system (feature ‘Code’ in Table 1) was developed, consisting of four parts separated by punctuation marks (‘.’ and ‘-‘) (Fig 4 & Table 2).

**Table 2:** Details of the coding system developed for the segregation of skeletal remains based on their degree of completeness. (Note: “-“indicates that the given category is not applicable; examples for Part 3 are provided according to the system illustrated in Fig 4).

Part 1: Level of completeness		Part 2: Presence (S) or absence (NS) of the skull	Part 3: Extra information on available or missing elements	Part 4: Serial number of the skeletal unit
*Code*	*meaning*			
COM	Complete (4 limbs)	S or NS	e.g., B3 Left radius missing	e.g., 412
INC	Incomplete (1–3 limbs)	S or NS	e.g., A123.D23 Right arm and Right tibia and fibula missing	e.g., 531
HSK	Half Skeleton (vertebral connection)	S or NS	UP = upper limb(s) present LOW = lower limb(s) present	e.g., 164
PAR	Body part	S or NS	e.g., B123 Left arm present	e.g., 012
SKU.ISO	Isolated skull	–	–	e.g., 229

The first part of the code, represented by the first 3–6 letters, indicates the level of completeness of the skeletal unit. Five subcategories were distinguished: 1) COM – complete skeletons with all four limbs present; 2) INC – incomplete skeletons missing 1–3 limbs but still assignable to a single individual; 3) HSK – half skeletons lacking continuity at some point along the vertebral column; 4) PAR – isolated parts, ranging from single bones to complete articulated limbs; and 5) SKU.ISO – skulls with no connection to postcranial elements. This classification primarily focuses on long bones, skulls, and hip bones, as they provide the most information for anthropological analyses [33]. The system is based on the assumption that two non-adjacent elements connected through a third, commonly adjacent bone belong to the same individual. Consequently, ribs and vertebrae are assumed to be present to some extent in the COM and INC categories. Only skeletons with both humeri and both femora present are classified as COM, as distal or unconnected limb bones are often difficult to associate with a single individual.

The second part of the code indicates whether a skull is associated with the unit: S for skull present, NS for skull not present. For isolated skulls, this information is already included in Part 1 and is therefore not applicable.

The third part encodes missing long bones in complete or incomplete units. In order to find an informative, yet easy-to-understand solution, the skeleton is divided into four sections: upper right (A), upper left (B), lower left (C), and lower right (D). Within each section, the most proximal element (humerus or femur) is numbered 1; distal and medial elements (ulna or tibia) are numbered 2; and distal and lateral elements (radius or fibula) are numbered 3 (Fig 4). For half skeletons or isolated postcranial elements, where the majority of the bones is missing, this part indicates which bones are present. This way, HSK.UP would represent the upper half of a skeleton, HSK.LOW would be used to code a skeleton incomplete at some point in the vertebral column, for which only the lower limbs are present. Isolated postcranial elements would receive a PAR code followed by the alphanumeric code part representing the present elements (PAR.C123 code for a left femur, tibia, and fibula). For isolated skulls, other code parts are not applicable.

The final part is a set of digits representing the serial number assigned to the skeletal unit during exhumation. If elements from two serial numbers are reassociated, the number of the first-discovered unit is used, and the original serial number of the second-discovered unit is recorded in the traceability field. In such cases, a change in the code category (e.g., from INC to COM) can occur when the amount of present elements after reassociation allows it following the above-mentioned rules.

**Fig 5:**
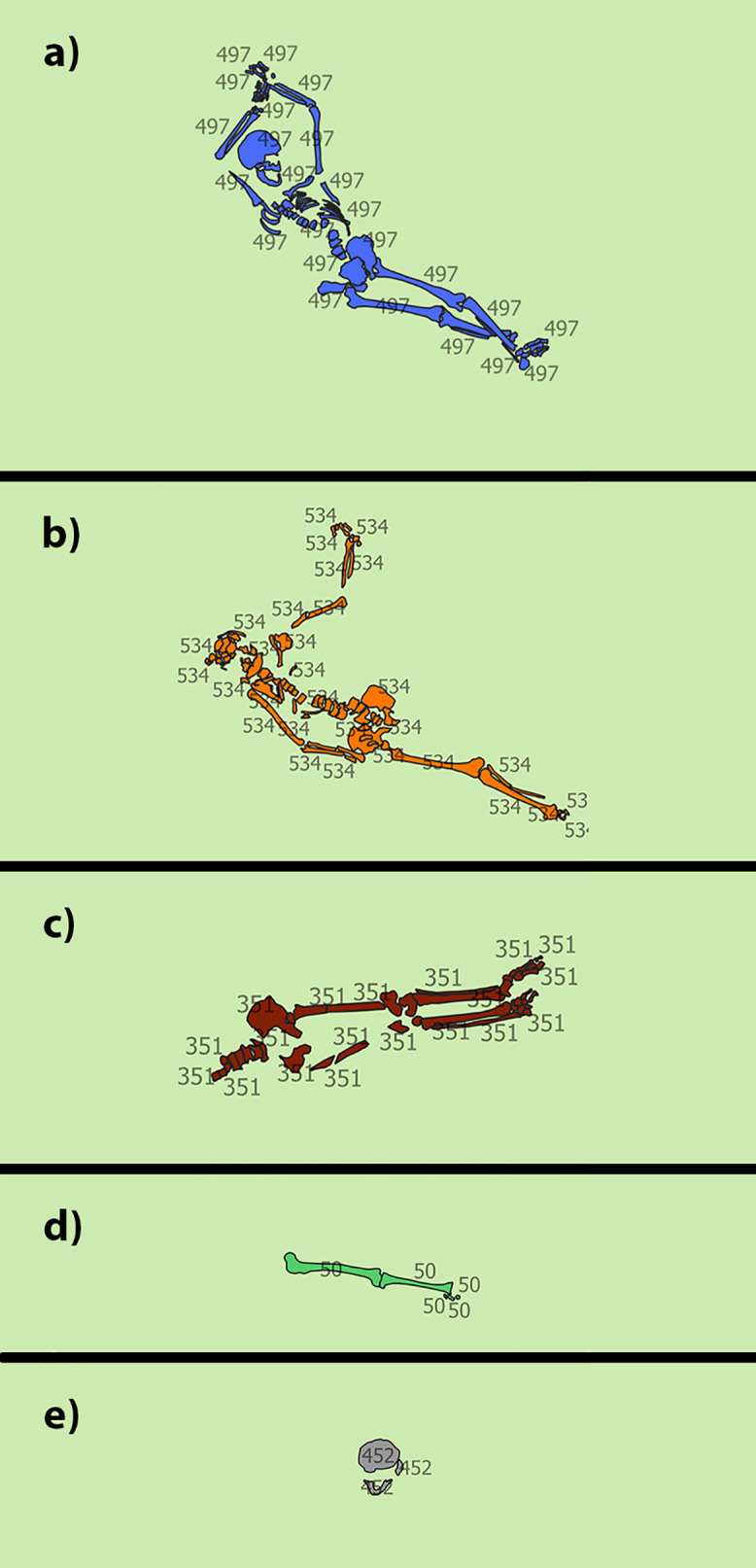
Examples of the different code groups visualized in QGIS 3.20.2: a) complete skeleton (COM.S-497); b) incomplete skeleton (INC.S.C123-543); c) half of a skeleton (HSK.NS.LOW-351) where continuity of skeletal elements is lost at the vertebral column; d) lower limb with no other skeletal element associated (PAR.NS.D123-50); and e) an isolated skull (SKU.ISO-452).

Following this system, “COM.S-497” describes a complete skeleton with associated skull, originally recorded as ID 497 (Fig 5a). “INC.S.C123-534” represents an incomplete skeleton with skull, missing the left femur, left tibia, and left fibula; originally recorded as ID 534 (Fig 5b). “HSK.NS.LOW-351” refers to a skeleton recovered to a certain point of the vertebral column, without a skull, and lower limbs present; originally recorded as ID 351 (Fig 5c). While “PAR.NS.D123-50” is a partial unit without skull, consisting only of an articulated right lower limb (femur, tibia, and fibula) (Fig 5d). Lastly, “SKU.ISO-452” (Fig 5e) is the code given to a skull with no connection to any other mayor skeletal element.

## Results

**Fig 6:**
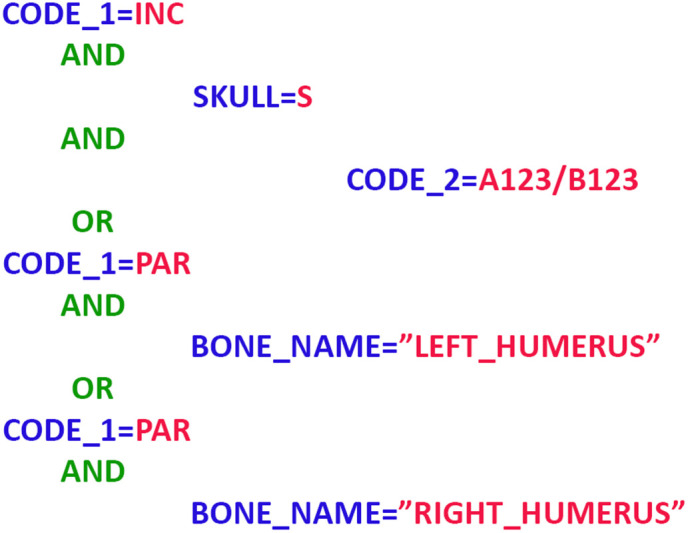
Example of code lines introduced in QGIS 3.20.2 for the segregation of cases missing both upper limbs (A123/B123), and the right and left upper limbs not associated to other skeletal elements (PAR; “LEFT_HUMERUS”; PAR; “RIGHT_HUMERUS”).

**Fig 7:**
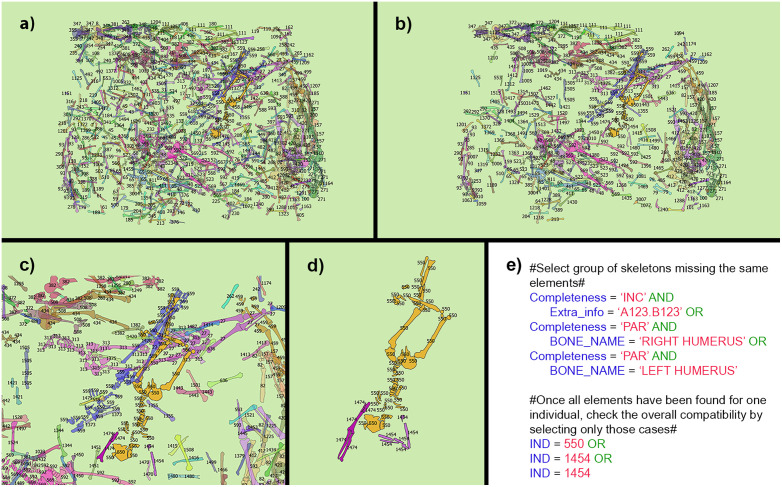
Reassociation of skeletons from MMG3 missing both upper limbs (INC.A123.B123): a) shows the cases with no filter for the compatible humeri, displaying all of them with no distinction between those that are already associated to other individuals, and those that are not; b) after applying selection filter, so that only humeri from both sides that are not already associated to an individual are shown; c) close up of case INC.S.A123.B123-550 for which two upper limbs are located in compatible locations (PAR.A123-1474, PAR.B123-1454); d) selected elements resulting from the visual assessment of the disposition of remains (note that when the ID number is selected, the totality of the skeletal elements registered to that ID number are shown, in this example, in addition to the most proximal elements, namely humeri, the remaining elements from the upper limbs are also displayed; and e) step-by-step reassociation as it would appear in QGIS 3.20.2 filter tool.

Following the step-by-step recording of data introduced in the Methods section (Table 1 & Fig 3), the QGIS 3.20.2 software presents the potential matches of elements based on their location and recorded attributes. On the individual level, results are obtained by writing a few lines of codes: the program will immediately collect and show those cases that meet the inserted criteria (e.g., skeletons missing a right ulna), in combination with the search of potentially matching missing elements (e.g., isolated right ulnae). For instance, given all skeletal sets that would include the skull, the axial skeleton as well as both upper limbs and the right lower limb (coded as INC.S.C123-XXX), would be missing the elements of the left lower limb. In this case, the left femora would be the key element, as it is the most proximal element among the ones that are missing, and therefore it must be the first one to be targeted. By inserting the coding presented in Fig 6, the software instantly displays all left femora that are currently not associated to any other individual. In comparison, avoiding the introduced coding system, the visualization would be much more difficult as the program would present all left femora regardless of whether they have already been associated to other individuals, making any attempt of visual assessment a longer and more challenging task, since it would show a varying amount of unnecessary data (Fig 7a).

This way, by applying the proposed filtering tools, in a matter of minutes, a single individual or all those individuals who are sharing the code “INC.S.A123/B123-XXX” and that are missing both upper limbs (i.e., humeri) would be analysed and their potential matches, that would only include those elements that are not yet associated with any other major unit, detected (Fig 7). Another example, crucial when dealing with battle-related mass graves such as MMG3, is the presentation and analysis of potentially isolated skulls and skeletons with no skulls. Finding the potential matches or excluding any association provides valuable assist for investigations aiming to better understand the perimortem traumas of the individuals buried in the grave.

The code and its structure are generalized and can be reutilised in basically every other case involving any combination, as only parts appearing in red on Fig 6 are to be adapted.

On the populational level, the results of the reassociation process with the current methodology is summarized in Table 3 & Table 4. In the case of MMG3, before attempting to reunite any element (Table 3), only around one-third (n = 99) of the total expected number of individuals was included in the complete (COM) category.

**Table 3:** Composition of MMG3 prior to the application of the methodology presented in this paper. The number (N) and percentage (%) are reported for each group according to the proposed coding system, distinguishing between bone sets with an associated skull (S), without an associated skull (NS), and their combined total.

	S		NS		TOTAL	
	N	%	N	%	N	%
COM	75.00	13.86	24.00	4.44	99.00	18.30
INC	92.00	17.01	58.00	10.72	150.00	27.73
SKU.ISO	116.00	21.44	0.00	0.00	116.00	21.44
HSK	17.00	3.14	63.00	11.65	80.00	14.79
PAR	15.00	2.77	81.00	14.97	96.00	17.74
TOTAL	315.00	58.23	226.00	41.8	541.00	100.00

**Table 4:** Composition of MMG3 after the application of the methodology presented in this paper. The number (N) and percentage (%) are reported for each group according to the proposed coding system, distinguishing between bone sets with an associated skull (S), without an associated skull (NS), and their combined total.

	S		NS		TOTAL	
	N	%	N	%	N	%
COM	125.00	26.94	41.00	8.84	166.00	35.78
INC	52.00	11.21	38.00	8.19	90.00	19.40
SKU.ISO	106.00	22.84	0.00	0.00	106.00	22.84
HSK	12.00	2.59	54.00	11.64	66.00	14.22
PAR	15.00	3.23	21.00	4.53	36.00	7.76
TOTAL	310.00	66.81	154.00	33.19	464.00	100.00

Using the methodology presented in this paper, on the other hand, resulted in an almost twofold increase in the number of complete skeletons (COM; n = 166) (Table 4), indicating that approximately 50% of the total expected number of individuals (n ≈ 320) could be analysed in their entirety. In addition, potential skull associations were identified for seven COM skeletons and three INC skeletons. Furthermore, seven HSK upper-body skeletons were observed in close spatial proximity to seven HSK lower-body skeletons, suggesting possible anatomical associations. The time required for this stage of the workflow – including the analysis of spatial relationships among all element groups, code assignment, and visual assessment of the results – was approximately two working days for a single team member.

## Discussion

The excavation, exhumation, and analysis of any form of plural burials constitute some of the most complex challenges encountered by biological and forensic anthropologists. The variability inherent in each grave has resulted in the development of predominantly case-specific methodologies [27,29,30], which limits the potential for broader comparative studies. A central issue in these investigations is the commingling of human remains. Numerous approaches have been proposed to address the reassociation of commingled elements [40,51]. However, both the level of commingling and the overall size of the assemblage fundamentally affect the scale of the challenge, as well as the efficiency and reliability of available methodological tools [33–35]. In this paper, we introduce a new integrative methodology designed to address a gap in the study of burials containing partially commingled remains from multiple individuals. To evaluate its potential and limitations, we discuss our results in the context of recurrent challenges encountered during the investigation of large-scale mass graves. MMG3 is a large, partially commingled assemblage – an appropriate sample to methodological tests, such as the one proposed in this paper.

Previous publications [36] have emphasized that the assemblage size substantially influences both the effectiveness and applicability of reassociation methods. Traditional approaches to the reassociation of commingled skeletal remains generally perform well in small assemblages; however, their effectiveness decreases as the assemblage size increases. Moreover, methods such as pair matching or articulation become impractical in large assemblages due to the exponentially increasing number of necessary comparisons, and the difficulty of segregating remains with limited morphological variations – a phenomenon more common in large, commingled contexts. While new matching and sorting tools have shown promising results [62], the equipment required may be too costly, and as of now, their applicability to the whole skeleton is yet to be tested. The methodology presented here is suitable for both small and large commingled assemblages, increasing the efficiency of reassociations when combined with other techniques. It provides extensive and accurate documentation within a relatively short time-frame and reduces potential damage to skeletal remains once exposed. Our experience indicates that a single person can manage the data-entry component of the method, allowing the rest of the team to focus on excavation and other documentation tasks (see [58,60]). Nonetheless, increasing the number of researchers responsible for data recording and drawing can substantially accelerate the excavation process [63].

As described earlier, our methodology incorporates the use of GIS software – a tool that has been applied to commingled assemblages and mass grave investigations before [49,50]; however, such approaches often require advanced proficiency in GIS and, in some cases, a strong statistical background. In contrast, our recommendations require only a basic knowledge of GIS technology and elementary skills on handling this type of software (e.g., creating polygon layers to highlight the different units and recording related attribute data) to perform the necessary tasks (see S1 File). We recommend QGIS as it is a freely available software that can be installed in most devices. Although a more detailed understanding of software functions and statistical principles is advantageous and can facilitate reassociation, neither is indispensable for applying the methodology proposed here.

The presented methodology functions as a visual analytical tool based on the assessment of spatial relationships among incomplete skeletal units and smaller articulated elements. Using control datasets (e.g., DNA analysis and time-lapse photography) in combination with GIS, the effectiveness and validity of approaches grounded in the hypothesis that anatomically compatible bones found in close proximity are likely to belong to the same individual have already been tested in previous studies [49,50]. Both the study by Tuller & Hofmeister [49], which investigated a late 20th-century conflict-related mass grave in Serbia, and the study by Voeller [50], which analysed an experimentally created mass grave using pig cadavers, reported promising results, with valid match rates of 88% and 91.89%, respectively.

In this context, the identification and reassociation of 166 complete skeletons – representing a 67.68% increase compared to the original 99 – along with seven half skeletons and 10 isolated skulls in MMG3 constitutes a realistic and robust outcome. Moreover, a substantial amount of analytical time was saved, particularly when considering the scale of the assemblage, which comprised more than 60,000 bones, and approximately 2,000 skeletal units of varying composition. It should be noted that both Tuller & Hofmeister [49] and Voeller [50] incorporated three-dimensional (3D) coordinates for reliable distance measurements. The inclusion of 3D spatial data – although not feasible in the case of MMG3 – has the potential to further enhance reassociation accuracy and/or reduce processing time, as comparisons across different stratigraphic layers could be conducted more efficiently.

The data presented here indicate that our methodology provides meaningful support in the investigation of mass graves, particularly due to its applicability in both field and laboratory settings, including storage and inventory management. When applied as an initial filtering tool during laboratory analysis, it can significantly reduce the number of required manual comparisons by visually highlighting only those cases where a potential match is possible, and by hiding remains already associated with other individuals (see Fig 2 and 7). At the same time, these tools serve as an additional test for identifying false positives from field determinations, thereby improving the accuracy and reliability of reassociation. Furthermore, the detailed codification system allows rapid interpretation of preservation status and inventory information directly on each storage unit (e.g., box, coffin, or casket), eliminating the need to re-examine the remains or repeatedly consult detailed descriptions, photographs, or colored skeletal silhouettes typically used to record preservation (e.g., [64]). This coding system could be modified as per needed given that the state of preservation of the target site differs from the burial examined in this article.

**Fig 8:**
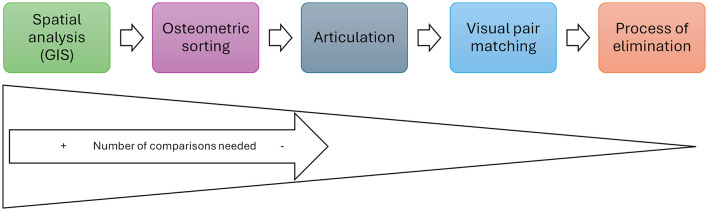
Diagram illustrating the stages of the proposed laboratory workflow. The first step consists of a visual assessment of the spatial relationships among incomplete elements. This step functions as a filtering process, whereby only elements in close proximity or sharing specific characteristics are subjected to further analysis.

**Fig 9:**
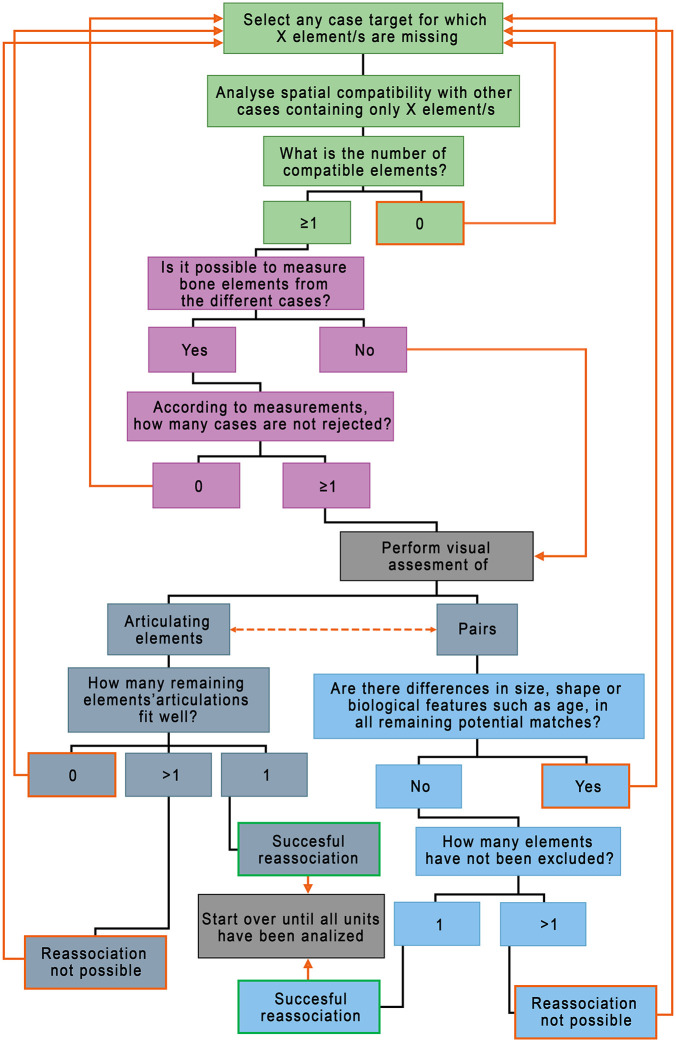
Algorithm for the reassociation of commingled human skeletal remains. In this step-by-step workflow, both DNA and process of elimination are ommitted as they are to be used separately. For secondary burial or ossuaries, osteometry should be the first step, as spatial relationships for two elements cannot guarantee belonging to a same individual. The visual assessment when both options are available starts with articulating elements as this is a less subjective technique, and continues with comparison between pairs. If the result of both examinations is satisfactory, a proposed match can be considered a successful reassociation.

Despite its advantages, the methodology has inherent limitations. In agreement with previous scholars [49,50], we consider it as part of a broader, integrative protocol in which additional analytical tools should be employed to achieve more comprehensive documentation and stronger evidentiary support for reassociation [36,65]. During laboratory analyses, the proposed approach can be effectively combined with established methods such as osteometric sorting, articulation, visual pair-matching, process of elimination, and, where feasible, DNA analysis, thereby forming an efficient and robust workflow (see Fig 8). An algorithm is proposed to help guide the process in a simple, concise and precise manner, where each iteration filters the potential matches first by analysing the spatial relationships, and then continues to further examine whatever elements remain by means of osteometric sorting and visual comparison – both articulation and pair matching – with the aim of excluding all those that present incompatible features. Ideally, a unique element would remain at the end of each process. Whenever more than one element, or none, survive the different tests, the reassociation cannot be confirmed. These actions are to be repeated as many times as needed until all elements have been analysed (Fig 9).

The presented tools were developed based on the partially commingled assemblage of MMG3. Nevertheless, they were designed to be applicable to a broad range of contexts, particularly primary, unaltered mass graves, multiple burials, and assemblages exhibiting varying degrees of commingling. However, factors such as bone preservation and the high degree of commingling may influence its applicability and effectiveness. In particular, poor preservation – where bone identification and assessments of anatomical compatibility are hindered – may substantially reduce the analytical potential. Importantly, the tools presented here have not yet been tested on completely commingled assemblages in which no anatomical connections are recorded in the field, and only limited utility should be expected in such cases. In addition to commingled human skeletal remains, the applicability of this method in cases involving zooarchaeological, paleontological, or paleoanthropological remains is yet to be tested.

Although circumstances commonly encountered during excavations involving human remains (e.g., time constraints or adverse environmental conditions) may occasionally prevent the complete recording of data in the software while in the field, we strongly recommend finalizing this step on site, whenever possible, particularly in assemblages with fragmented remains, as fragmentation may substantially reduce the possibilities for reassociation and subsequent laboratory analyses. Field-based data entry improves accuracy and facilitates the resolution of potentially conflicting cases during excavation. Nonetheless, our experience shows that the remaining tasks can be successfully completed in the laboratory, and our results demonstrate significant increase in the number of reassociated remains suitable for further anthropological analysis (see Tables 3 and 4).

## Conclusions

The methodological approach applied in the investigation of the partially commingled skeletal remains from MMG3 further demonstrates that the analysis of spatial relationships among skeletal elements in large-scale assemblages – where at least some anatomical connections are presumed – is an efficient and practical tool [49,50].

Incorporating spatial analysis as an initial filtering step in the reassociation process significantly reduces the number of comparisons required in subsequent analytical stages, thereby decreasing overall processing time and improving methodological efficiency when combining with already existing techniques (e.g., osteometric sorting). The approach proposed in this study – including the coding system and the direct recording of observed features within the software environment – establishes a standardized analytical framework that facilitates future comparative studies. At the same time, it remains flexible, allowing straightforward modifications and adaptations to accommodate the specific conditions and constraints of individual projects.

Finally, to better understand the applicability and limitations of this methodological approach across different archaeological contexts, it should be tested in a wider range of cases exhibiting diverse characteristics. These include variations in grave size, number of individuals, and degree of commingling. Therefore, our aim is to conduct further exploratory research on commingled human skeletal remains – particularly studies integrating GIS technology and applied statistical methods.

## Supporting information

S1 File Recording of skeletal elements using Quantum Geographic Information System (QGIS). (PDF)
