# Supplementary material for: New integrative methodology for the excavation and study of mass graves and partially commingled remains
Source: PLoS One. 2026 Jul 17;21(7):e0353420. doi: 10.1371/journal.pone.0353420 (PMC13378973; doi:10.1371/journal.pone.0353420)
Supplement: S1 File — (PDF) [file pone.0353420.s001.pdf]

S1 Text: Recording of skeletal elements using Quantum Geographic Information System (QGIS)

The recording of skeletal elements in QGIS 3.20.2 requires a series of simple steps that can be completed by any person regardless of whether they have prior experience using GIS, and QGIS in particular. A series of prerequisites, equally easily achievable, include the addition of the different georeferenced orthophotos taken during the fieldwork documentation as "Raster Layers". Once the photographic documentation has been inserted in QGIS 3.20.2, the recording process can be initiated.

Firstly, the specialist should choose a case. In Supplementary Figure 1, a skull, labelled in the field as case "12" is perfectly visible.

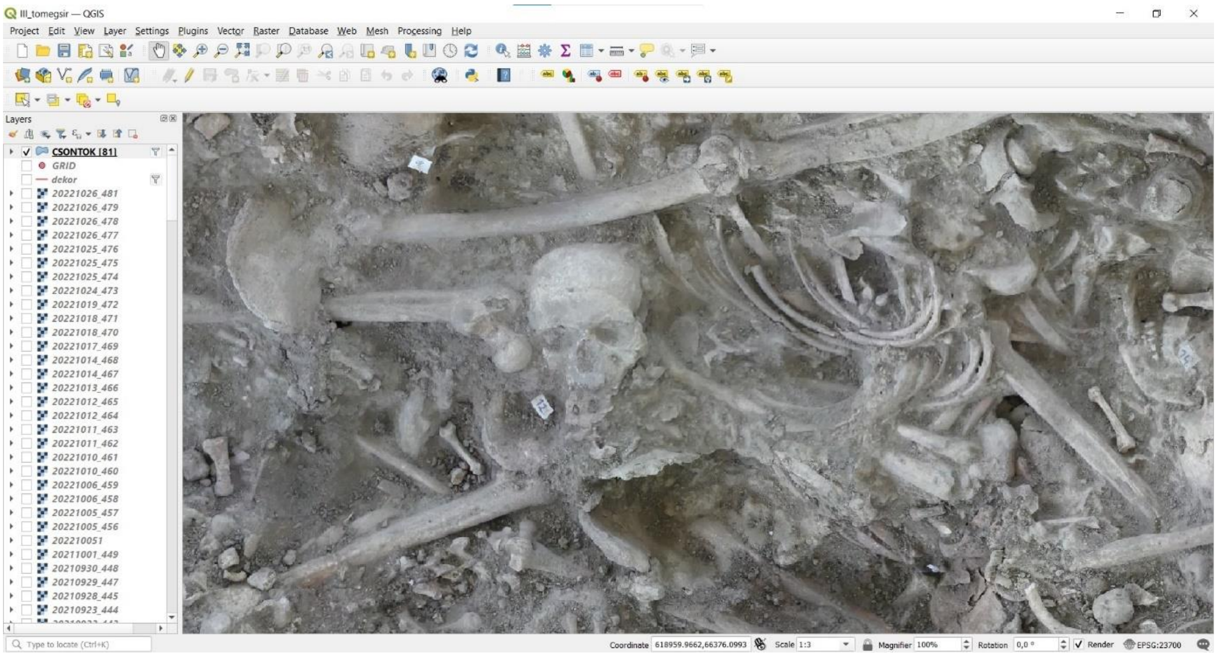

Supplementary Figure 1. Case selection in QGIS 3.20.2. In this screenshot case 12 (marked in the field with a label) consist solely of a skull.

Once the case has been chosen, the next step is to create a polygon layer over the photography that shows that case. In order to complete this step, the specialist must click in "Toggle Editing" (Supplementary Figure 2) and then "Add Polygon Feature" (Supplementary Figure 3).

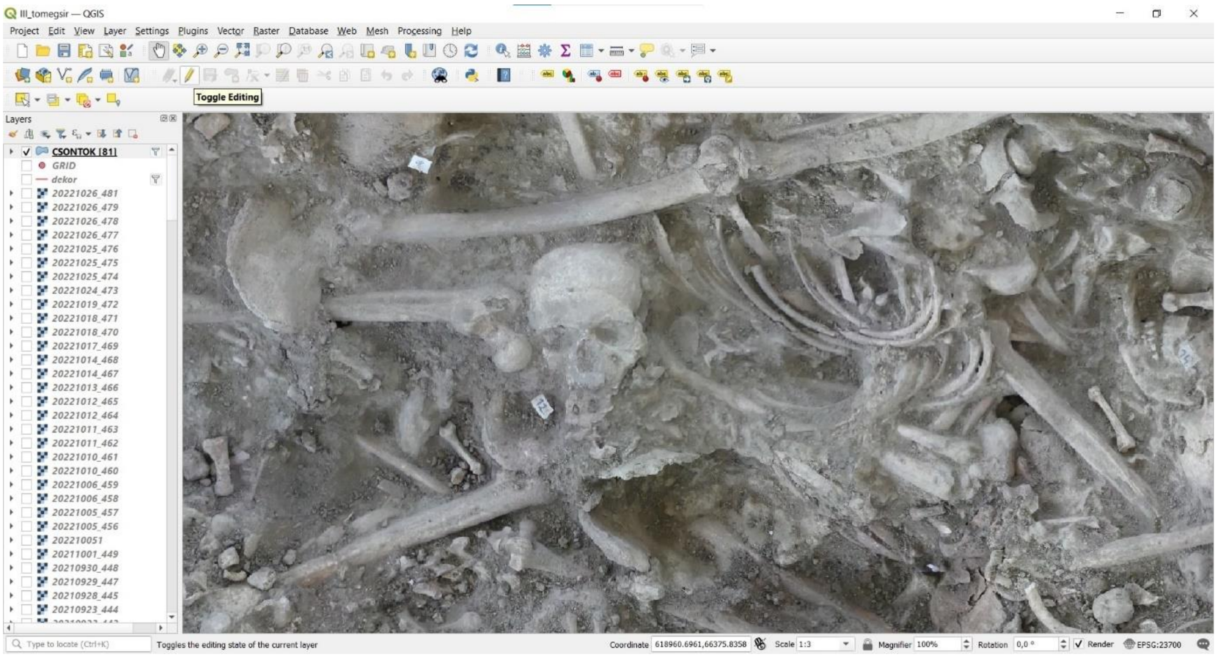

Supplementary Figure 2. Selection of "Toggle Editing" in QGIS 3.20.2.

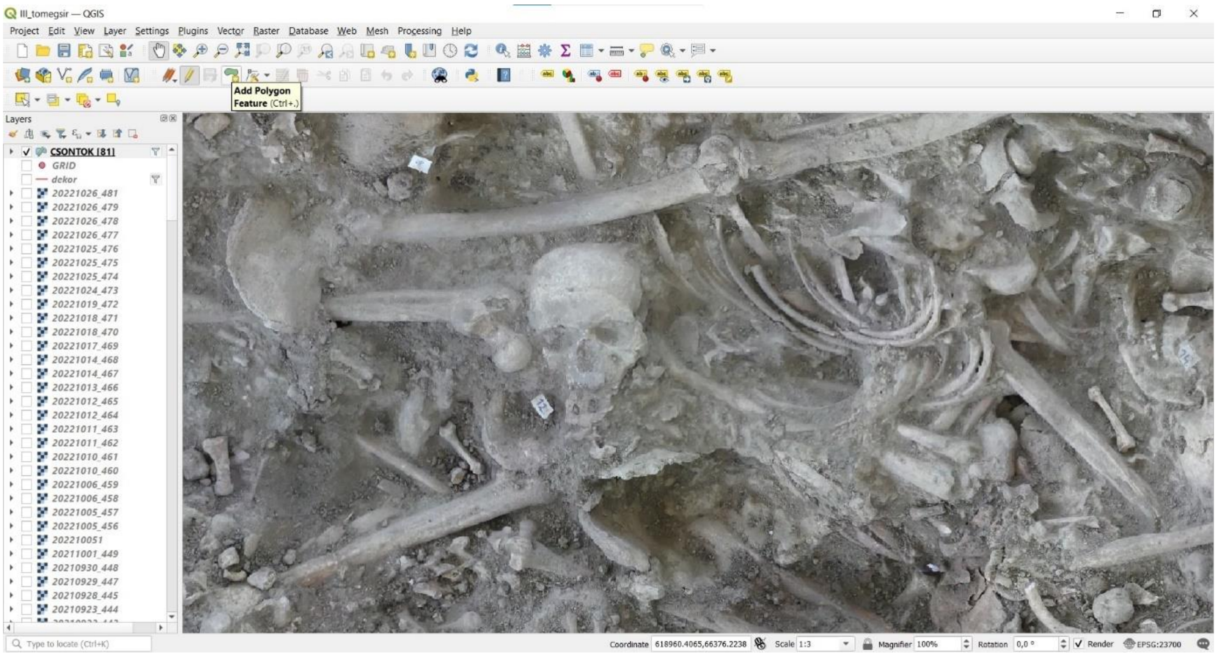

Supplementary Figure 3. Selection of "Add Polygon Feature" in QGIS 3.20.2.

A polygon is a succession of lines that represent objects in a 2-dimensional plane. For this reason, a series of lines will have to be drawn around the perimeter of the selected element. With every left click a new point will be fixed and a line will be drawn from that point to the previous one. Polygons are to be finalized at the same point where they were started. (Supplementary Figures 4 and 5).

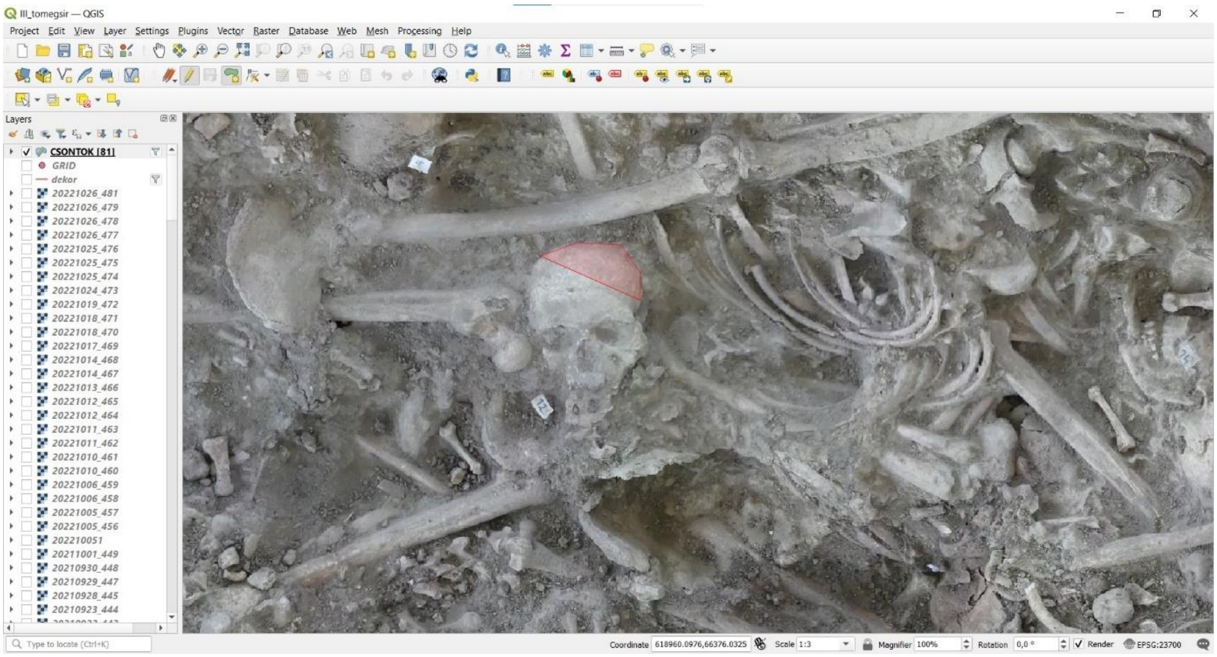

Supplementary Figure 4. Creation of new polygon layer in QGIS 3.20.2. Drawing a polygon over skull "12".

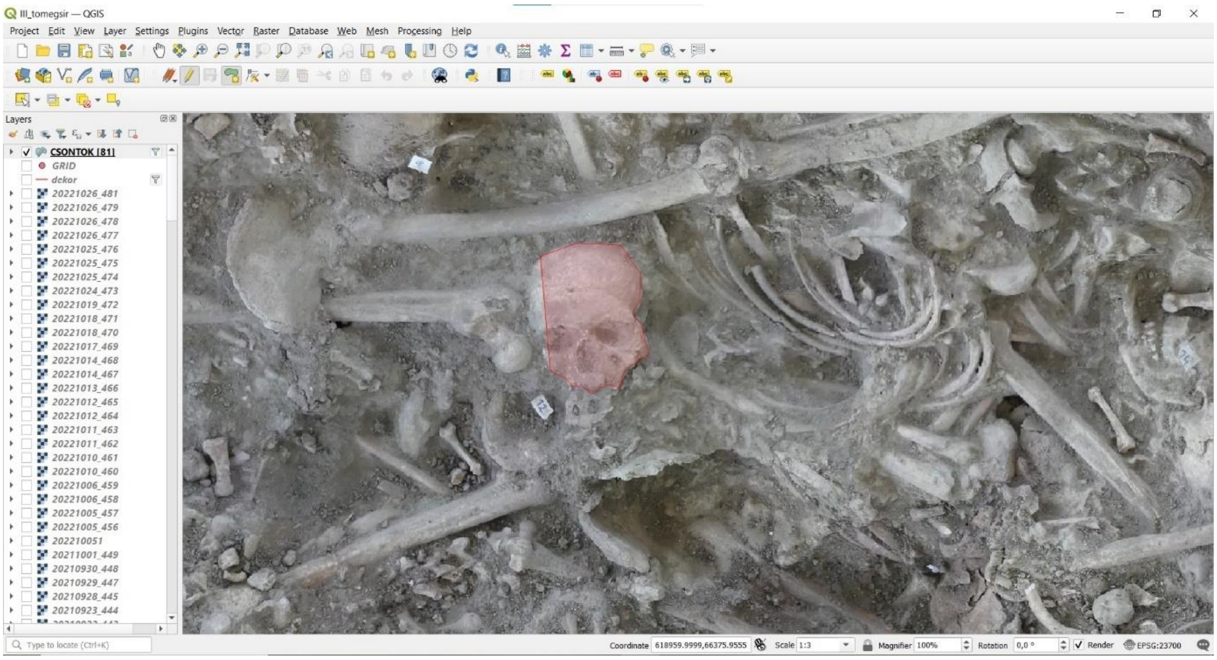

Supplementary Figure 5. Creation of new polygon layer in QGIS 3.20.2. Closing polygon over the photography of element "12".

Once the polygon is completed, the specialist must click right on the mouse to close the polygon and open the "Feature Attribute" table (Supplementary Figure 6). All the fields listed in the "Feature Attribute" table are to be filled with all the information available for the specialist (Supplementary Figure 7).

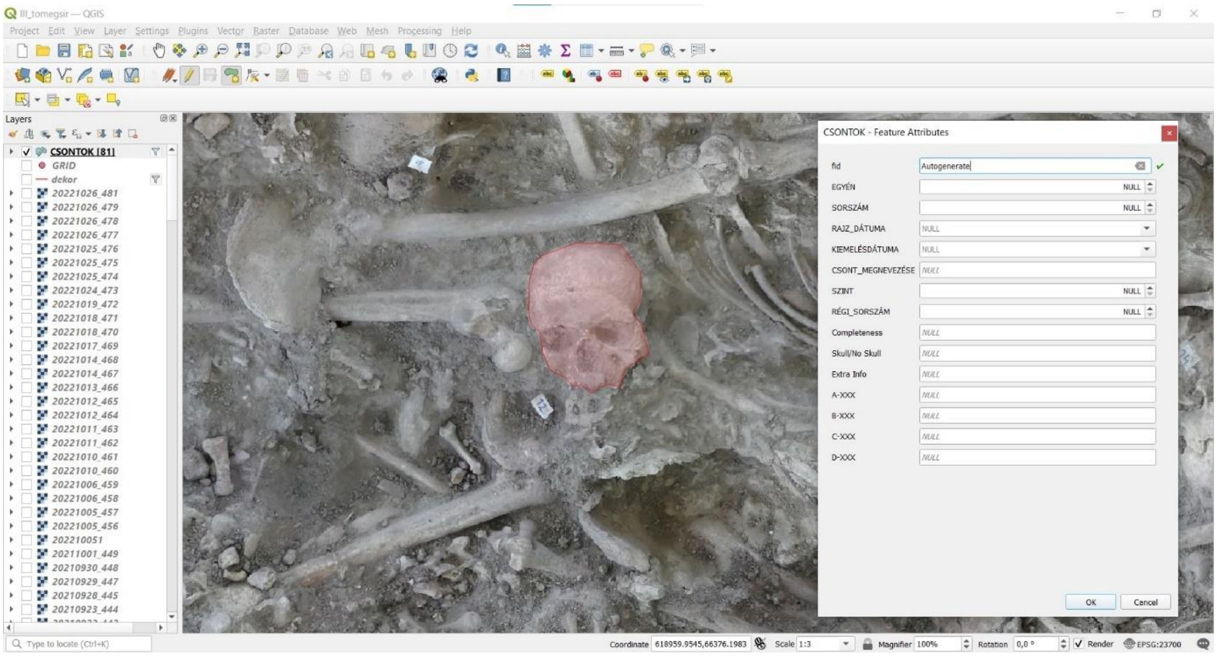

Supplementary Figure 6. Creation of new polygon layer in QGIS 3.20.2. Polygon completion and "Feature Attribute" table.

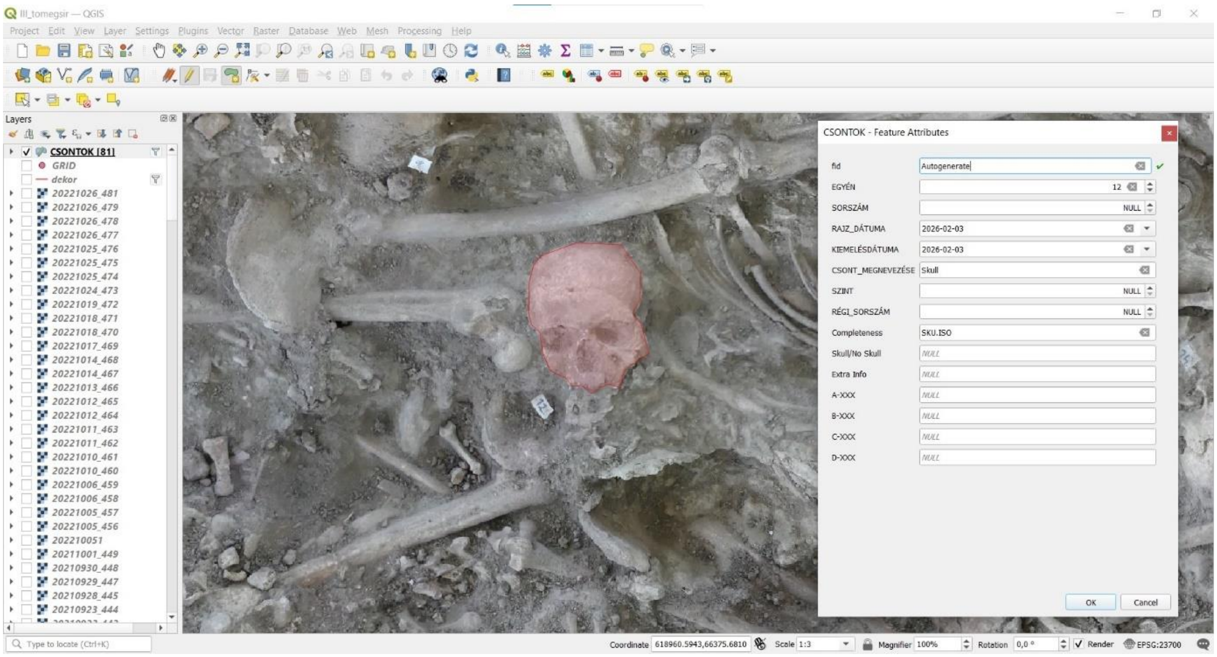

Supplementary Figure 7. Creation of new polygon layer in QGIS 3.20.2. Data recorded in "Feature Attribute" table.

After recording the necessary information in the attribute table, and clicking "OK", the new element will appear, and the information recorded will be available to be apply following the methodology proposed in the main text (Supplementary Figure 8).

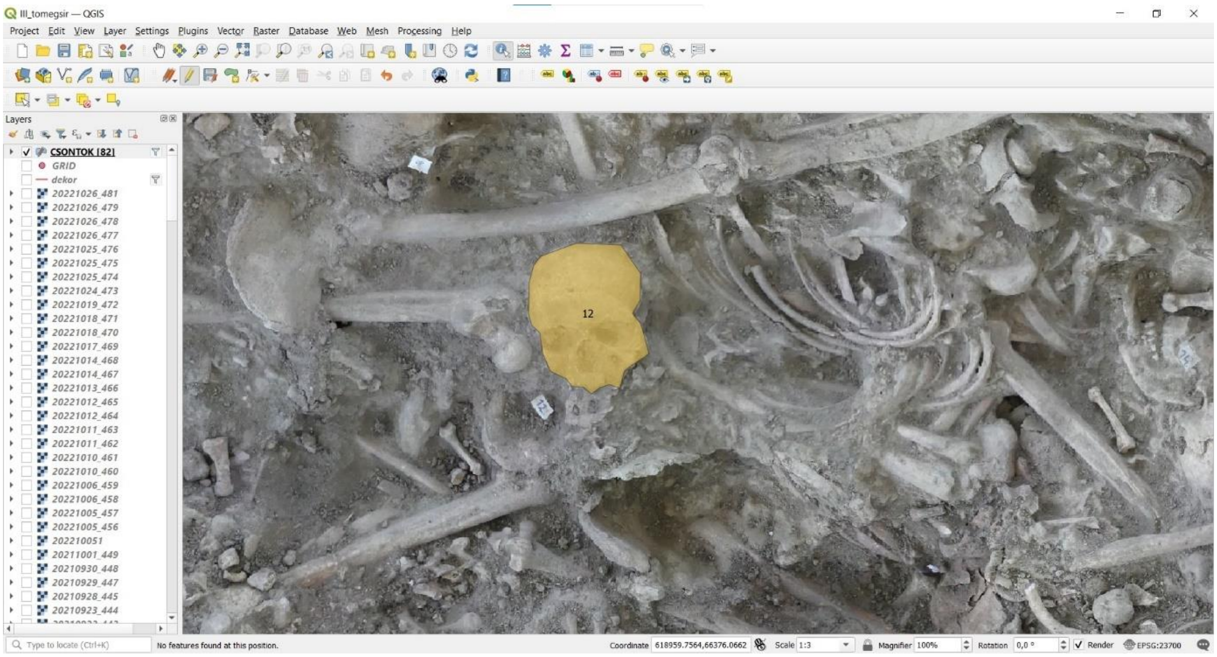

Supplementary Figure 8. Creation of new polygon layer in QGIS 3.20.2. Element "12" now represented as a coloured polygon with its information recorded and ready to be used.
