# Supplementary material for: New integrative methodology for the excavation and study of mass graves and partially commingled remains
Source: PLoS One. 2026 Jul 17;21(7):e0353420. doi: 10.1371/journal.pone.0353420 (PMC13378973; doi:10.1371/journal.pone.0353420)
Supplement: Attachment — Submitted filename: Response to Reviewers.docx [file pone.0353420.s004.docx]

Dr. Mario Novak, PhD

Academic Editor

PLOS ONE

Dear Dr. Novak,

We are very grateful for your and the reviewers’ insightful and constructive comments regarding our manuscript entitled “*New Integrative Methodology for the excavation and study of mass graves and partially commingled remains*” that was submitted to PLOS ONE (manuscript ID: PONE-D-26-10119). The main text has been modified following the suggestions. The revised files have been uploaded to the submission site of the journal. In addition, Journal Requirement number 3 demanded a modification on the financial disclosure, we modified it as requested, however we would like to highlight that the new statement is only partially accurate as the funding mentioned in the original form of our financial disclosure only refers to an Open Access fund, granted by the University of Szeged to the corresponding author. The funder provided support in the form of salaries for several authors [MAM, LK, OS, VV, RK, BT and GP], but did not have any additional role in the study design, data collection and analysis, decision to publish, or preparation of the manuscript. We are sure that you and the reviewers helped us to improve the quality of our manuscript.

**Response to the Academic Editor’s comments**

1) **This is an excellent paper that substantially adds to our efforts in the recovery and analysis of human remains from both, forensic and archaeological contetxs. The authors provide a new, improved methodology on how to successfully recover and study such contexts. I agree with both reviewers that this is a very important piece without major flaws. However, as R2 indicated the authors are trying maybe a bit too much to make this methodology universal and applicable to all possible cases which might not always be the case.**

We would like to thank the Academic Editor for the positive evaluation and constructive comments on our work. We agree that this method is not universal, and we would like to emphasize that it was never our intention to present it in this manner. We are fully aware of its limitations and the fact that it can only be applied successfully in cases meeting certain criteria. This was indicated in the Discussion section (lines 527–536):

“*This method can be applied across a wide range of contexts, including primary unaltered mass graves/multiple burials and those exhibiting varying degrees of commingling. However, factors such as bone preservation and the high degree of commingling may influence its applicability and effectiveness. In particular, poor preservation – where bone identification and assessments of anatomical compatibility are hindered – may substantially reduce the analytical potential. Importantly, the tools presented here have not yet been tested on completely commingled assemblages in which no anatomical connections are recorded in the field, and only limited utility should be expected in such cases. In addition to commingled human skeletal remains, the applicability of this method in cases involving zooarchaeological, paleontological, or paleoanthropological remains is yet to be tested.*”

We consider the application of GIS technology to be a substantial aid in the reassociation process as it can compensate for the lost efficacy of other techniques– such as osteometry or visual comparisons of articulating or paired bones– when dealing with large assemblages.

However, we do not support the idea that GIS technology should replace existing tools such as osteometric sorting or visual comparison of articulating elements and paired bones. On the contrary, we support their combined use, as stated in the Discussion section (lines 499–505):

“*Despite its advantages, the methodology has inherent limitations. In agreement with previous scholars [49,50], we consider it as part of a broader, integrative protocol in which additional analytical tools should be employed to achieve more comprehensive documentation and stronger evidentiary support for reassociation [36,65]. During laboratory analyses, the proposed approach can be effectively combined with established methods such as osteometric sorting, articulation, visual pair-matching, process of elimination, and, where feasible, DNA analysis, thereby forming an efficient and robust workflow (see Fig 8).*”

Our proposal is therefore based on integrating the described techniques and methods into a broader and structured workflow intended to facilitate the work of specialists facing similar situations. We attempted to summarize this workflow in Fig. 9:

“*Algorithm for the reassociation of commingled human skeletal remains. In this step-by-step workflow, both DNA and process of elimination are ommitted as they are to be used separately. For secondary burial or ossariums, osteometry should be the first step, as spatial relationships for two elements cannot guarantee belonging to a same individual. The visual assessment when both options are available starts with articulating elements as this is a less subjective technique and continues with comparison between pairs. If the result of both examinations is satisfactory, a proposed match can be considered a successful reassociation.*”

To make these ideas clearer, we propose the following changes in the revised version of the manuscript:

In the discussion section, lines 454–456 of the revised version of our manuscript, we have inserted the following sentence:

Original version (lines 453–454):

*“The methodology presented here is suitable for both small and large commingled assemblages.”*

Revised version (lines 454–456):

*“The methodology presented here is suitable for both small and large commingled assemblages, increasing the efficiency of reassociations when combined with other techniques”*

2) **Additionaly, this method also requires a certain technical background that might not be available to all those dealing with such burials.**

Regarding the technical skills required to apply this methodology effectively, we agree with both the Editor and Reviewer#2 that a certain level of technical knowledge is necessary to navigate the software and conduct the proposed analyses. However, precisely because only a basic level of GIS proficiency is required, we consider this to be an advantage compared to other approaches that also incorporate GIS technology (e.g., Tuller&Hofmeister, 2014; Voeller, 2024). To make the methodology accessible even to readers without prior experience using GIS software, we included a Supplementary File covering the most fundamental operations required to record and manage data using georeferenced photographs of the site, which are already common practice in archaeological documentation.

In agreement with the comment from Reviewer#2, we modified lines 462–471 in the revised version of the manuscript to emphasize that a certain level of knowledge is indeed required:

Original text (460–467):

*“As described earlier, our methodology incorporates the use of GIS software – a tool that has been applied to commingled assemblages and mass grave investigations before [49,50]; however, such approaches often require advanced proficiency in GIS and, in some cases, a strong statistical background. In contrast, our recommendations allow any specialist with basic GIS skills (e.g., creating polygon layers to highlight the different units and recording related attribute data) to perform the necessary tasks (see Supplementary File 1). Although a more detailed understanding of software functions and statistical principles is advantageous and can facilitate reassociation, neither is indispensable for applying the methodology proposed here.”*

Revised text (lines 462–471):

*“As described earlier, our methodology incorporates the use of GIS software – a tool that has been applied to commingled assemblages and mass grave investigations before [49,50]; however, such approaches often require advanced proficiency in GIS and, in some cases, a strong statistical background. In contrast, our recommendations require only a basic knowledge of GIS technology and elementary skills on handling this type of software (e.g., creating polygon layers to highlight the different units and recording related attribute data) to perform the necessary tasks (see Supplementary File 1). We recommend QGIS as it is a freely available software that can be installed in most devices. Although a more detailed understanding of software functions and statistical principles is advantageous and can facilitate reassociation, neither is indispensable for applying the methodology proposed here.”*

References:

Tuller H, Hofmeister U. Spatial Analysis of Mass Grave Mapping Data to Assist in the Reassociation of Disarticulated and Commingled Human Remains. In Adams BJ, Byrd J, editors. Commingled Human Remains.: Academic Press; 2014. p. 7-32.

Voeller S. The use of GIS for cases of comingling. In Ross AH, Byrd JH, editors. Methodological and Technological Advances in Death Investigations.: Academic Press; 2024. p. 225-246.

3) **Therefore, I would suggest that the authors maybe 'tone' down a bit certain parts of the text. There are also some other minor suggestions and comments by both reviewers that I think would improve the manuscript in certain points.**

We appreciate this suggestion and have revised the relevant sections of the manuscript to clarify our position and avoid overstating the universality of the proposed methodology. In addition, we have incorporated the remaining comments and corrections suggested by both reviewers throughout the revised version of the manuscript. Furthermore, we corrected two words that were wrongly spelled or misused (lines 423 and 527), these are indicated with a comment reading “Typo corrected”. Please find our detailed response to the Reviewers’ comments below.

**Response to the Reviewers’ comments**

**Reviewer #1:**

1. **In following, there are some suggestions of changes and rephrasing in the in general well written and interesting paper. None of suggested is vital, overall recommendation is “minor revisions”**

We would like to thank Reviewer for the positive evaluation of our manuscript, as well as for the constructive comments and suggestions that helped us improve the clarity and quality of the paper.

2. **Abstract: rephrase the “reliable results”. I deeply hope, there are no analyses aiming to provide any other, but reliable results. Sound weird.**

We thank the Reviewer for this comment and agree that, in principle, analyses should aim to provide reliable results. We have therefore removed the word “reliable” from line 27 in the revised version of the manuscript.

3. **Abstract: rephrase “recovered”. There is NO WAY to increase the number of recovered skeletons. You only can increase the number of identified ones…**

We thank the Reviewer for this comment too and fully agree with it. We have replaced the word “recovered” with “individualized” in line 35 of the revised version of our manuscript.

**Main text:**

4. **Lines 100-108: please rephrase, this is surely true, but NOT only for plural burials, even if there it makes the huge difference.**

We appreciate the indication and agree that the variables listed in the original text may affect the excavation of any burial context, although they are particularly relevant in plural burials. We therefore rephrased the text as follows:

Original text:

*“Distinct variables can potentially complicate the excavation and exhumation of remains interred in plural burials [31].*”

Revised text:

“*Distinct variables can potentially complicate the excavation and exhumation of remains interred in any burial context, especially those containing multiple individuals [31].*”

5. **Lines 246-253: ethics is nowadays doubtlessly a relevant topic, even for human remains of >500 years of age, however, it is not a part of the main publication body. Unless Plos has a new way of treating it? Please, move to the end or to the beginning, outside the main text body.**

We thank the Reviewer for this comment. However, this section was added following a request from the editorial office.

6. **Line 291: rather 00001 to 99999, isn’t it? Otherwise you’re going to have a problem with any programmes from Microsoft.**

We thank the Reviewer for their comment on this matter. Since we mainly worked within QGIS, this issue did not arise during our analyses. However, we agree that if the dataset is exported to other software (e.g., Microsoft Excel), the absence of leading zeros may indeed create complications. Therefore, we have included this suggestion into line 291 of the revised manusctipt.

**Fig 3.:**

7. **Bone type in full names? LHu/RHu for right and left humerus would do and, on your thousands of lines, save a lot of data**

We are grateful for this suggestion and agree that reducing the number of characters inserted during data recording can improve efficiency. However, throughout our project we consistently used full names, and the figures presented in the manuscript were generated directly from our original QGIS dataset. To preserve consistency with the original workflow, we retained the full names in the examples presented.

Additionally, one advantage of the proposed system is the relatively short length of the coding lines used within the QGIS filters, as represented in Figure 6. Consequently, the difference in character length between full names and abbreviations would not substantially affect the complexity of the coding process. Nevertheless, we agree that abbreviations are entirely feasible, and therefore added the following clarification to the subsection “Bone type and laterality” in Table 1: “*This can be the bone’s full name or any unique abbreviation to each element.”*

8. **Possible connection: “=” implies, there is FOR SURE a connection, being “the same”. We cannot say this FOR SURE by any osteology alone, without DNA, this is always merely “99.9%”. Rather some marking such as “≈” or “→”**

We thank the Reviewer for this observation and agree that a different symbol would better represent a proposed rather than definitive association. Accordingly, we replaced the “=” symbol with “*→”* in the subsection “Possible connection with other elements” of Table 1.

9. **Miscellaneous: is it reasonable to mix up information directly relevant to identification of individuals (such as age-at-death, sex, systemic pathologies) with interesting but not relevant for sorting information (such as cut marks or osteomyelitis)? Then, this information misses their main purpose, to identify the missing parts, and go on to another topic, the investigation of the individuals themselves. This is not your goal here, otherwise, the system would need a lot of other info, too…**

We truly appreciate this insightful comment. Although recording such information may initially appear impractical due to the additional time and space required, our experience with Mass Grave No.3 at the Mohács National Memorial Site demonstrated that characteristics such as biological sex, age-at-death, pathological changes, or perimortem trauma can indeed play a significant role in reassociation.

In our assemblage, the majority of individuals were identified as adult males. Consequently, remains belonging to females or subadults became highly distinctive and therefore particularly useful for segregation purposes. The same logic applies to pathological changes and perimortem trauma: although some pathological changes are relatively common, severe lesions are comparatively rare, while cut marks –especially in a context where decapitation represents one of the working hypotheses– may also serve as potentially segregating features.

The purpose of the “Miscellaneous” category was therefore to create a flexible field that can accommodate characteristics considered especially informative within a given assemblage, without requiring the systematic recording of all non-diagnostic features. In our case, for example, only females and subadults were specifically noted, whereas the category was intentionally left blank for adult males by default. We believe this approach allows future users to adapt the system according to the specific characteristics and research priorities of their own assemblages.

10. ***Line 303ff: I miss any information on hands and feet. A difficult topic, not really possible to solve to 100% in a mass grave, but very important for the “injuries” topic! Do you score them at all? Do you score them separately somewhere else? If yes, where and how? Please specify. And, if not scored yet, set this as a new project direction…*😉 *Without the bones of hands and feet, it would be really easy to use abbreviations (R/L + Hu, Fe, Ul, Ra, Ti, Fi)***

We are very grateful for this insight and fully agree that the reassociation of hand and foot bones represents both a particularly challenging and highly relevant aspect of the analysis.

Given that these elements belong to the smallest and most labile articulations of the human skeleton, their recovery and reassociation are especially difficult in plural burials and commingled assemblages. In the case of Mass Grave No. 3 at the Mohács National Memorial Site the successful recovery rate of these elements during excavation was rather limited. In many cases, they were found disarticulated or displaced, frequently even inside skulls. Furthermore, reassociation is complicated by the relative lack of methods (e.g., osteometric) specifically designed for these elements.

Considering these, we examined hand and foot bones separately. Nevertheless, if their spatial position is accurately recorded during excavation, and they can reasonably be expected to be found relatively close to their original depositional context, they may still contribute to spatial analyses. However, as noted above, the reassociation of these elements remains methodologically limited due to the lack of specialized analytical tools. We fully agree with the Reviewer that this topic deserves dedicated future research and holds considerable methodological potential.

11. **Lines 421-429: Great! Impressive improvement of effectiveness!**

We sincerely thank the Reviewer for this positive feedback. We are encouraged by the results obtained during our project and look forward both to apply the methodology to other sites and to hear from colleagues who may find the approach useful in their own work.

12. **Lines 531-534: completely commingled assemblages would need a related, but very different approach. For now, let’s stick to such non-commingled or just slightly commingled contexts, such as yours presented here. This is already helpful enough!**

We are grateful for this comment and agree that completely commingled assemblages require a substantially different methodological approach. As stated in the “Discussion” section (lines 528–537 in the original manuscript):

“*This method can be applied across a wide range of contexts, including primary unaltered mass graves/multiple burials and those exhibiting varying degrees of commingling. However, factors such as bone preservation and the high degree of commingling may influence its applicability and effectiveness. In particular, poor preservation – where bone identification and assessments of anatomical compatibility are hindered – may substantially reduce the analytical potential. Importantly, the tools presented here have not yet been tested on completely commingled assemblages in which no anatomical connections are recorded in the field, and only limited utility should be expected in such cases. In addition to commingled human skeletal remains, the applicability of this method in cases involving zooarchaeological, paleontological, or paleoanthropological remains is yet to be tested.*”

Please note that this paragraph has been modified following comments by Reviewer#2, however, we believe it still highlights the need for a different course of action in case of completely commingled assemblages, it now reads:

*“The presented tools were developed based on the partially commingled assemblage of MMG3. Nevertheless, they were designed to be applicable to a broad range of contexts, particularly primary, unaltered mass graves, multiple burials, and assemblages exhibiting varying degrees of commingling. However, factors such as bone preservation and the high degree of commingling may influence its applicability and effectiveness. In particular, poor preservation – where bone identification and assessments of anatomical compatibility are hindered – may substantially reduce the analytical potential. Importantly, the tools presented here have not yet been tested on completely commingled assemblages in which no anatomical connections are recorded in the field, and only limited utility should be expected in such cases. In addition to commingled human skeletal remains, the applicability of this method in cases involving zooarchaeological, paleontological, or paleoanthropological remains is yet to be tested.”*

We strongly recommend this methodological approach primarily for cases meeting the criteria outlined above. At the same time, we emphasize the need for particular caution when dealing with extremely commingled assemblages or ossuary contexts, as also noted in the legend of Figure 9:

“*Fig 9. Algorithm for the reassociation of commingled human skeletal remains. In this step-by-step workflow, both DNA and process of elimination are ommitted as they are to be used separately. For secondary burial or ossariums, osteometry should be the first step, as spatial relationships for two elements cannot guarantee belonging to a same individual. The visual assessment when both options are available starts with articulating elements as this is a less subjective technique, and continues with comparison between pairs. If the result of both examinations is satisfactory, a proposed match can be considered a successful reassociation.*”

We would like to express our gratitude to Reviewer**#**1 for their valuable comments and suggestions.

**Reviewer #2:**

1. **“In general, this article gives a great method for reassociation with commingled remains. I am a firm believer that there are multiple ways to approach commingled remains, and that there is no single solution to how to approach them in terms of analysis or interpretation. The method described within provides another tool in the skillset that may be utilized by practitioners, and therefore is worthy of publication.**

**I agree that there is a lack of consistent terminology used for commingled remains, and that this is a result of different training and the relative youth of the study of commingled remains as a focus. Until around the 1990s, commingled remains were typically relegated to appendices or stored without significant study since the assemblages were thought to be too difficult or not to produce significant results. It is bound to be the case that consistent methodology would not be forthcoming.**

**They’re probably also correct that the time is ripe for developing terminological consistency, much as Buikstra and Ubelaker did in 1994 for general bioarchaeological recording. I’m not sure that an article like this is the place to provide that, since they cannot approach all situations that produce commingled remains. I think that type of volume will be coming in the next few years though, and work such as this article will help to formulate those guidelines.**

We are truly grateful for these comments on our work and agree on the necessity of developing consistent terminology and methodological approaches for the examination of commingled human remains.

2. **I feel that the authors are very committed to their method and see it as the best approach to resolve commingling and assist in reassociation. It looks great, but it isn’t a universal fix and the authors should acknowledge that there are different approaches that may be employed to good results.”**

We thank the Reviewer for this important comment and agree that our methodological proposal is not a universal solution. In the original version of our manuscript, we acknowledged that the methodology presented here has inherent limitations (lines 500–513). Therefore, we intentionally avoided referring to it as “universal” or the “best solution” to the problem.

In agreement with previous studies (Tuller&Hofmeister, 2014; Voeller, 2024), we support the use of GIS technology for the analysis of spatial relationships in partially commingled assemblages, especially in large-scale contexts. Our proposal aims to integrate GIS-based spatial analysis with other well-established methods such as osteometric sorting and visual comparison of articulating or paired elements (represented in Fig .9 of the original manuscript), thereby compensating for the reduced effectiveness of these techniques when the number of potential matches becomes very large.

However, in order to further emphasize this important point, we have rephrased the relevant parts in the revised version of the manuscript. We hope that the revised text more clearly reflects our position on this matter.

In the discussion section, lines 454–456 of the revised version of our manuscript, we have modified the original phrasing:

Original version (lines 453–454):

*“The methodology presented here is suitable for both small and large commingled assemblages.”*

Revised version (lines 454–456):

*“The methodology presented here is suitable for both small and large commingled assemblages, increasing the efficiency of reassociations when combined with other techniques”*

References:

Tuller H, Hofmeister U. Spatial Analysis of Mass Grave Mapping Data to Assist in the Reassociation of Disarticulated and Commingled Human Remains. In Adams BJ, Byrd J, editors. Commingled Human Remains.: Academic Press; 2014. p. 7-32.

Voeller S. The use of GIS for cases of comingling. In Ross AH, Byrd JH, editors. Methodological and Technological Advances in Death Investigations.: Academic Press; 2024. p. 225-246.

3. **“This method relies on a significant amount of technological expertise, including using QGIS. QGIS may not be available to all researchers and/or all researchers may not have the technological know-how to utilize this method. This is the primary issue that I have with this article, otherwise, the method seems sound and the supplementary text is well done and provides a step-by-step process to employ the authors’ methodology.”**

We thank the Reviewer for their valuable comment on the technological skills required to apply the proposed methodology. We have firsthand experience with the difficulties associated with applying methods that require prior technical knowledge, and therefore we do not wish to downplay this aspect of our proposal.

We acknowledge that some level of expertise with GIS technology, and this software in particular, is necessary, as reflected in line 464 of the original version of the manuscript:

“*In contrast, our recommendations allow any specialist with basic GIS skills (e.g., creating polygon layers to highlight the different units and recording related attribute data) to perform the necessary tasks (see Supplementary File 1)*”

At the same time, previous methodological studies demonstrated that the analysis of commingled remains is inherently complex and often benefits from the application of techniques that are more sophisticated than those typically required for standard anthropological analyses of complete and individualized skeletons. We consider the technical requirements of our proposal substantially less demanding than those associated with other GIS-based approaches for commingled assemblages (e.g., Tuller&Hofmeister, 2014; Voeller, 2024), and regard this as an advantage of our methodology.

In addition, QGIS is freely available software that can be installed on most modern computers, as its hardware requirements in terms of memory, storage space, processor, and graphics capacity are relatively modest. Although QGIS is a powerful and complex tool overall, the specific operations required for our workflow are comparatively simple if researchers have a proper guidance. This was the idea behind the detailed step-by-step guide provided in the Supplementary File 1. To clarify this aspect further, we modified the relevant paragraph in the revised version of the manuscript as follows:

original version of our manuscript (lines 460–467):

“*As described earlier, our methodology incorporates the use of GIS software – a tool that has been applied to commingled assemblages and mass grave investigations before [49,50]; however, such approaches often require advanced proficiency in GIS and, in some cases, a strong statistical background. In contrast, our recommendations allow any specialist with basic GIS skills (e.g., creating polygon layers to highlight the different units and recording related attribute data) to perform the necessary tasks (see Supplementary File 1). Although a more detailed understanding of software functions and statistical principles is advantageous and can facilitate reassociation, neither is indispensable for applying the methodology proposed here.*”

Revised version of our manuscript (lines 462–471):

“*As described earlier, our methodology incorporates the use of GIS software – a tool that has been applied to commingled assemblages and mass grave investigations before [49,50]; however, such approaches often require advanced proficiency in GIS and, in some cases, a strong statistical background. In contrast, require only a basic knowledge of GIS technology and elementary skills in handling this type of software (e.g., creating polygon layers to highlight different units and recording related attribute data) to perform the necessary tasks (see Supplementary File 1). We recommend QGIS as it is a freely available software that can be installed in most devices. Although a more detailed understanding of software functions and statistical principles is advantageous and can facilitate reassociation, neither is indispensable for applying the methodology proposed here.*”

References:

Tuller H, Hofmeister U. Spatial Analysis of Mass Grave Mapping Data to Assist in the Reassociation of Disarticulated and Commingled Human Remains. In Adams BJ, Byrd J, editors. Commingled Human Remains.: Academic Press; 2014. p. 7-32.

Voeller S. The use of GIS for cases of comingling. In Ross AH, Byrd JH, editors. Methodological and Technological Advances in Death Investigations.: Academic Press; 2024. p. 225-246.

4. **The case study used here has a good number of articulated elements. I think if they frame their work such that it is good for commingled remains where there is significant articulation, it would be good. Fragmentation complicates this picture immensely. I don’t suggest that the authors attempt to apply their methodology to cases of fragmented and commingled remains, but since fragmentation also generally accompanies commingling, they should specify which types of assemblages their method would be best applied to. If they choose to address this, they should identify how such methodology would be utilized on extreme processing assemblages from the American Southwest, for example (e.g., Kuckelman, Lightfoot, and Martin 2000; Martin, Akins, and Toll 2014; Martin and Osterholtz 2020).**

We are thankful for this comment and acknowledge that the fragmentation issue was not discussed in detail in the original version of the manuscript. Although Mass Grave No. 3 at the Mohács National Memorial Site exhibits a certain degree of fragmentation and a relatively low level of articulation (only approximately one-third of the estimated 300 individuals were originally recovered in full articulation), we recognize that many published assemblages present substantially poorer preservation and much higher degrees of disarticulation.

As we have not yet had the oppurtinity to test the proposed method under such extreme conditions, our conclusions regarding other sites remain hypothetical. Nevertheless, we believe the method may still prove useful provided that the underlying assumption remains valid – namely, that anatomically compatible skeletal elements found in close spatial proximity are more likely to belong to the same individual. This assumption has previously been explored by both Tuller & Hofmeister, and Voeller.

If this premise holds true, spatial analysis may still aid in the reassociation of highly fragmented remains by facilitating fragment conjoining and narrowing the number of potential matches requiring further analyses. In the case of Mass Grave No. 3 at the Mohács National Memorial Site, this approach proved highly beneficial, and we hope to evaluate its applicability in additional contexts in future research.

Following the Reviewer’s suggestion, we provided further clarification in the revised manuscript discussing the influence of preservation and fragmentation on the applicability of the method.

Original version (lines 537–543):

*“Although circumstances commonly encountered during excavations involving human remains (e.g., time constraints or adverse environmental conditions) may occasionally prevent the complete recording of data in the software while in the field, we strongly recommend finalizing this step on site.”*

Revised version (lines 546–554):

*“Although circumstances commonly encountered during excavations involving human remains (e.g., time constraints or adverse environmental conditions) may occasionally prevent the complete recording of data in the software while in the field, we strongly recommend finalizing this step on site, whenever possible, particularly in assemblages with fragmented remains, as fragmentation may substantially reduce the possibilities for reassociation and subsequent laboratory analyses.”*

5. **I like the numbering and identification system that the authors propose, but I think this is more effective for a commingled situation with more complete elements.**

We sincerely thank the Reviewer for these insights regarding the proposed numbering and identification system. The system has, so far, only been applied to Mass Grave No. 3 at the Mohács National Memorial Site, which naturally limits our ability to evaluate its effectiveness in assemblages with substantially different preservation conditions.

Nonetheless, the coding system was intentionally designed to remain flexible and adaptable to more fragmentary contexts. As mentioned in the response to the previous comment, additional fields can easily be incorporated when necessary, allowing skeletal regions –instead of complete bones– to be recorded at varying levels of detail, ranging from diaphysis/epiphysis division to specific zones or landmarks.

We believe that such modifications would still remain compatible with the spatial relationships analyses proposed in our workflow.

Accordingly, we have included the following sentence in lines 502–503 in the revised version of the manuscript:

“*This coding system could be modified as needed depending on the preservation state of the assemblage under investigation*.”

6. **Generally, I like this article quite a bit. It’s well founded and provides a great case study. I think the authors should reframe it a bit to indicate that it is a method that works well with a particular type of commingled assemblage. Once that is done, I think this will be a great contribution to the wide-ranging body of literature that has come out in the past 20 years on commingling.**

We are grateful to Reviewer **#**2 for their positive assessment of our work and gladly followed their suggestions to further clarify that we do not present this methodology as a universal solution. Rather, we propose it as a methodological contribution that may assist specialists dealing with the challenges posed by plural burials, mass graves, and partially commingled assemblages. We hope that the Reviewer will find the revisions made to the manuscript satisfactory.

7. ***One general note: MNI is determined, not estimated. Since MNI is a measure of the observed number of elements (or features/zones), it is determined. MLNI is estimated, though. I’d caution the authors to be careful in their language.***

We genuinely appreciate this comment and have modified the term utilized when referring to the Minimum Number of Individuals.

We would like to sincerely thank Reviewer#2 for their valuable comments and suggestions on our work.

**Response to the** **Journal Requirements**

**When submitting your revision, we need you to address these additional requirements.**

1. **Please ensure that your manuscript meets PLOS ONE's style requirements, including those for file naming. The PLOS ONE style templates can be found at**

[**https://journals.plos.org/plosone/s/file?id=wjVg/PLOSOne_formatting_sample_main_body.pdf**](https://journals.plos.org/plosone/s/file?id=wjVg/PLOSOne_formatting_sample_main_body.pdf) **and**

[**https://journals.plos.org/plosone/s/file?id=ba62/PLOSOne_formatting_sample_title_authors_affiliations.pdf**](https://journals.plos.org/plosone/s/file?id=ba62/PLOSOne_formatting_sample_title_authors_affiliations.pdf)

Thank you for this reminder. We have ensured that the revised manuscript complies with all PLOS ONE style requirements, including those related to file naming.

2. **In your manuscript, please provide additional information regarding the specimens used in your study. Ensure that you have reported human remain specimen numbers and complete repository information, including museum name and geographic location.**

**If permits were required, please ensure that you have provided details for all permits that were obtained, including the full name of the issuing authority, and add the following statement:**

**'All necessary permits were obtained for the described study, which complied with all relevant regulations.'**

**If no permits were required, please include the following statement:**

**'No permits were required for the described study, which complied with all relevant regulations.'**

**For more information on PLOS One's requirements for paleontology and archeology research, see** [**https://journals.plos.org/plosone/s/submission-guidelines#loc-paleontology-and-archaeology-research**](https://journals.plos.org/plosone/s/submission-guidelines#loc-paleontology-and-archaeology-research)**.**

Regarding the specimens analyzed in this study, all existing information on the human skeletal remains, including repository details, the name of the institution, and its geographic location, is provided in the manuscript. Human remains specimen numbers were not included as the skeletal material is highly fragmented and commingled, making it impossible at the current stage to assign individual specimen numbers. This will only be possible after the reassociation process has been completed, which is the topic of the current manuscript.

No permits were required for the analyses conducted as part of this study. Accordingly, the statement “No permits were required for the described study, which complied with all relevant regulations” was already included in line 252 of the original manuscript and has been retained in the revised version.

3. **Thank you for stating the following financial disclosure:**

**“This study was carried out within the framework of the Mohács 500 project, with the support of the Ministry of Agriculture of the Hungarian Government (government decree nos. 1159/2023., IV. 27. and 1288/2024., IX. 19.), awarded to GP. This work was also funded by the University Research Scholarship Programme – Cooperative Doctoral Programme 2024 (2024-2.1.2-EKÖP-KDP) of the Hungarian Ministry of Culture and Innovation (grant agreement no. EKÖP-KDP-24-SZTE-2) to MAM, by the University of Szeged Open Access Fund (grant agreement no. 8439) to MAM, and by the STARTING_25 sub-programme of the National Research Excellence Programme, under the management of the Hungarian National Research, Development and Innovation Office (grant agreement no. STARTING 153194) to OS. The funders had no role in the study design; in the collection, analysis, and interpretation of data; in the writing of the manuscript; and in the decision to submit the manuscript for publication.”**

**We note that one or more of the authors is affiliated with the funding organization, indicating the funder may have had some role in the design, data collection, analysis or preparation of your manuscript for publication; in other words, the funder played an indirect role through the participation of the co-authors. If the funding organization did not play a role in the study design, data collection and analysis, decision to publish, or preparation of the manuscript and only provided financial support in the form of authors' salaries and/or research materials, please do the following:**

**1. Review your statements relating to the author contributions, and ensure you have specifically and accurately indicated the role(s) that these authors had in your study. These amendments should be made in the online form.**

**2. Confirm in your cover letter that you agree with the following statement, and we will change the online submission form on your behalf:**

We confirm that the funder provided support in the form of salaries for authors [MAM, LK, OS, VV, RK, BT and GP], but did not have any additional role in the study design, data collection and analysis, decision to publish, or preparation of the manuscript. The specific roles of these authors are articulated in the ‘author contributions’ section.

4. **We note that Figures 1 and 3 and supplementary figures 1 - 8 in your submission contain copyrighted images. All PLOS content is published under the Creative Commons Attribution License (CC BY 4.0), which means that the manuscript, images, and Supporting Information files will be freely available online, and any third party is permitted to access, download, copy, distribute, and use these materials in any way, even commercially, with proper attribution. For more information, see our copyright guidelines: http://journals.plos.org/plosone/s/licenses-and-copyright.**

**We require you to either (1) present written permission from the copyright holder to publish these figures specifically under the CC BY 4.0 license, or (2) remove the figures from your submission:**

**1. You may seek permission from the original copyright holder of Figure(s) [#] to publish the content specifically under the CC BY 4.0 license.**

**We recommend that you contact the original copyright holder with the Content Permission Form (http://journals.plos.org/plosone/s/file?id=7c09/content-permission-form.pdf) and the following text:**

**“I request permission for the open-access journal PLOS ONE to publish XXX under the Creative Commons Attribution License (CCAL) CC BY 4.0 (http://creativecommons.org/licenses/by/4.0/). Please be aware that this license allows unrestricted use and distribution, even commercially, by third parties. Please reply and provide explicit written permission to publish XXX under a CC BY license and complete the attached form.”**

**Please upload the completed Content Permission Form or other proof of granted permissions as an "Other" file with your submission.**

**In the figure caption of the copyrighted figure, please include the following text: “Reprinted from [ref] under a CC BY license, with permission from [name of publisher], original copyright [original copyright year].”**

We have obtained the necessary permissions from the copyright holder to publish the relevant figures under the CC BY 4.0 license. The corresponding permission documentation has been uploaded to the submission system as (Permission_DeAndres_et_al_Figures_1; Permission_DeAndres_et_al_Figures_2; Permission_DeAndres_et_al_Figures_3).

In addition, we have revised the captions of Figures 1 and 3 and Supplementary Figures S1–S8 to include the required copyright and permission statements in accordance with the journal’s guidelines.

5. **We note that Figure 1 in your submission contain map images which may be copyrighted. All PLOS content is published under the Creative Commons Attribution License (CC BY 4.0), which means that the manuscript, images, and Supporting Information files will be freely available online, and any third party is permitted to access, download, copy, distribute, and use these materials in any way, even commercially, with proper attribution. For these reasons, we cannot publish previously copyrighted maps or satellite images created using proprietary data, such as Google software (Google Maps, Street View, and Earth). For more information, see our copyright guidelines: http://journals.plos.org/plosone/s/licenses-and-copyright.**

**We require you to either (1) present written permission from the copyright holder to publish these figures specifically under the CC BY 4.0 license, or (2) remove the figures from your submission:**

**1. You may seek permission from the original copyright holder of Figure(s) [#] to publish the content specifically under the CC BY 4.0 license.**

**We recommend that you contact the original copyright holder with the Content Permission Form (http://journals.plos.org/plosone/s/file?id=7c09/content-permission-form.pdf) and the following text:**

**“I request permission for the open-access journal PLOS ONE to publish XXX under the Creative Commons Attribution License (CCAL) CC BY 4.0 (http://creativecommons.org/licenses/by/4.0/). Please be aware that this license allows unrestricted use and distribution, even commercially, by third parties. Please reply and provide explicit written permission to publish XXX under a CC BY license and complete the attached form.”**

**Please upload the completed Content Permission Form or other proof of granted permissions as an "Other" file with your submission.**

**In the figure caption of the copyrighted figure, please include the following text: “Reprinted from [ref] under a CC BY license, with permission from [name of publisher], original copyright [original copyright year].”**

We appreciate the opportunity to clarify the source and licensing status of the maps presented in Figure 1.

The caption of Figure 1 in the original manuscript states:

“Fig 1. a) Map of Hungary and location of the Mohács National Memorial Site (image obtained from OpenStreetMap and OpenStreetMap Foundation, which is made available under the Open Database License (https://www.openstreetmap.org/copyright)); b) The location of Mass Grave No. 3 within the Mohács National Memorial Site (image obtained from OpenStreetMap and OpenStreetMap Foundation, which is made available under the Open Database License (https://www.openstreetmap.org/copyright)); and c) Orthophoto of Mass Grave No. 3 of the Mohács National Memorial Site (source of the orthophoto: Photo Collection and Archaeological Database of the Janus Pannonius Museum, Pécs, Hungary).”

The maps shown in Figure 1a–b were created using data from OpenStreetMap, which is distributed under the Open Database License (ODbL) and is explicitly listed by PLOS ONE as an acceptable source for map data (<https://journals.plos.org/plosone/s/figures#loc-maps>). Accordingly, we believe that these figures comply with the journal’s copyright and licensing requirements. The required attribution has been included in the figure caption.

6. **Please include a copy of Table 1 which you refer to in your text on page 14.**

Thank you for bringing this to our attention. After carefully reviewing both the original and revised versions of the manuscript, we confirmed that Table 1 was included in the submitted manuscript and spans pages 12–14. The table remains in the same location in the revised version.

7. **We note you have included a table to which you do not refer in the text of your manuscript. Please ensure that you refer to Table 5 in your text; if accepted, production will need this reference to link the reader to the Table.**

Thank you for this comment. After carefully reviewing the manuscript, we were unable to identify a Table 5 in either the original or revised version of the submission. The manuscript contains only Tables 1–4. Therefore, we believe this comment may have been included in error, and no additional reference to Table 5 is required.

8. **If the reviewer comments include a recommendation to cite specific previously published works, please review and evaluate these publications to determine whether they are relevant and should be cited. There is no requirement to cite these works unless the editor has indicated otherwise.**

We have carefully reviewed all reviewer comments and found no recommendations to cite specific previously published works. The references mentioned by the reviewers were provided solely to support and contextualize their comments. Consequently, no additional references were added to the revised manuscript.

9. **Please review your reference list to ensure that it is complete and correct. If you have cited papers that have been retracted, please include the rationale for doing so in the manuscript text, or remove these references and replace them with relevant current references. Any changes to the reference list should be mentioned in the rebuttal letter that accompanies your revised manuscript. If you need to cite a retracted article, indicate the article’s retracted status in the References list and also include a citation and full reference for the retraction notice.**

We have carefully reviewed the reference list and verified that all citations are complete and accurate. We also confirmed that none of the cited publications have been retracted. Therefore, no modifications to the reference list were necessary.

In the revised version of our manuscript, we have tried to execute all suggestions of the reviewers. We hope this new version will be suitable for publication in PLOS ONE.

Thank you again for the reviewers’ insightful and constructive comments and your editorial work!

Yours sincerely,

Marcos De Andrés Montero

Corresponding author

Department of Biological Anthropology, University of Szeged

Közép fasor 52, H-6726, Szeged, Hungary

Email: [mdeandres1993@gmail.com](mailto:mdeandres1993@gmail.com)
