# Supplementary material for: New integrative methodology for the excavation and study of mass graves and partially commingled remains
Source: PLoS One. 2026 Jul 17;21(7):e0353420. doi: 10.1371/journal.pone.0353420 (PMC13378973; doi:10.1371/journal.pone.0353420)
Supplement: Attachment — Submitted filename: Plos One Review De Andres et al.docx [file pone.0353420.s002.docx]

Plos One Review De Andres et al. PONE-D-26-10119

In following, there are some suggestions of changes and rephrasing in the in general well written and interesting paper. None of suggested is vital, overall recommendation is “minor revisions”

Abstract: rephrase the “reliable results”. I deeply hope, there are no analyses aiming to provide any other, but reliable results. Sound weird.

Abstract: rephrase “recovered”. There is NO WAY to increase the number of recovered skeletons. You only can increase the number of identified ones…

Main text:

Lines 100-108: please rephrase, this is surely true, but NOT only for plural burials, even if there it makes the huge difference.

Lines 246-253: ethics is nowadays doubtlessly a relevant topic, even for human remains of >500 years of age, however, it is not a part of the main publication body. Unless Plos has a new way of treating it? Please, move to the end or to the beginning, outside the main text body.

Line 291: rather 00001 to 99999, isn’t it? Otherwise you’re going to have a problem with any programmes from Microsoft.

Fig 3.:

Bone type in full names? LHu/RHu for right and left humerus would do and, on your thousands of lines, save a lot of data

Possible connection: “=” implies, there is FOR SURE a connection, being “the same”. We cannot say this FOR SURE by any osteology alone, without DNA, this is always merely “99.9%”. Rather some marking such as “≈” or “→”

Miscellaneous: is it reasonable to mix up information directly relevant to identification of individuals (such as age-at-death, sex, systemic pathologies) with interesting but not relevant for sorting information (such as cut marks or osteomyelitis)? Then, this information misses their main purpose, to identify the missing parts, and go on to another topic, the investigation of the individuals themselves. This is not your goal here, otherwise, the system would need a lot of other info, too…

Line 303ff: I miss any information on hands and feet. A difficult topic, not really possible to solve to 100% in a mass grave, but very important for the “injuries” topic! Do you score them at all? Do you score them separately somewhere else? If yes, where and how? Please specify. And, if not scored yet, set this as a new project direction…😉 Without the bones of hands and feet, it would be really easy to use abbreviations (R/L + Hu, Fe, Ul, Ra, Ti, Fi)

Lines 421-429: Great! Impressive improvement of effectiveness!

Lines 531-534: completely commingled assemblages would need a related, but very different approach. For now, let’s stick to such non-commingled or just slightly commingled contexts, such as yours presented here. This is already helpful enough!
