# Supplementary material for: New integrative methodology for the excavation and study of mass graves and partially commingled remains
Source: PLoS One. 2026 Jul 17;21(7):e0353420. doi: 10.1371/journal.pone.0353420 (PMC13378973; doi:10.1371/journal.pone.0353420)
Supplement: Peer review history

# Peer review: Decision Letter 0

**DOI:** 10.1371/journal.pone.0353420.r001

**Authors:** Mario Novak

24 Apr 2026

PONE-D-26-10119

New Integrative Methodology for the excavation and study of mass graves and partially commingled remains

PLOS One

Dear Dr. De Andrés Montero,

Thank you for submitting your manuscript to PLOS ONE. After careful consideration, we feel that it has merit but does not fully meet PLOS ONE’s publication criteria as it currently stands. Therefore, we invite you to submit a revised version of the manuscript that addresses the points raised during the review process.

Please submit your revised manuscript by Jun 08 2026 11:59PM. If you will need more time than this to complete your revisions, please reply to this message or contact the journal office at plosone@plos.org. When you're ready to submit your revision, log on to https://www.editorialmanager.com/pone/ and select the 'Submissions Needing Revision' folder to locate your manuscript file.

Please include the following items when submitting your revised manuscript:

- A letter that responds to each point raised by the academic editor and reviewer(s). You should upload this letter as a separate file labeled 'Response to Reviewers'.
- A marked-up copy of your manuscript that highlights changes made to the original version. You should upload this as a separate file labeled 'Revised Manuscript with Track Changes'.
- An unmarked version of your revised paper without tracked changes. You should upload this as a separate file labeled 'Manuscript'.

If you would like to make changes to your financial disclosure, please include your updated statement in your cover letter. Guidelines for resubmitting your figure files are available below the reviewer comments at the end of this letter.

If applicable, we recommend that you deposit your laboratory protocols in protocols.io to enhance the reproducibility of your results. Protocols.io assigns your protocol its own identifier (DOI) so that it can be cited independently in the future. For instructions see: https://journals.plos.org/plosone/s/submission-guidelines#loc-laboratory-protocols. Additionally, PLOS ONE offers an option for publishing peer-reviewed Lab Protocol articles, which describe protocols hosted on protocols.io. Read more information on sharing protocols at https://plos.org/protocols?utm_medium=editorial-email&utm_source=authorletters&utm_campaign=protocols.

As the corresponding author, your ORCID iD is verified in the submission system and will appear in the published article. PLOS supports the use of ORCID, and we encourage all coauthors to register for an ORCID iD and use it as well. Please encourage your coauthors to verify their ORCID iD within the submission system before final acceptance, as unverified ORCID iDs will not appear in the published article. *Only*  the individual author can complete the verification step; PLOS staff *cannot*  verify ORCID iDs on behalf of authors.

We look forward to receiving your revised manuscript.

Kind regards,

Mario Novak

Academic Editor

PLOS One

Journal Requirements:

When submitting your revision, we need you to address these additional requirements.

1. Please ensure that your manuscript meets PLOS ONE's style requirements, including those for file naming. The PLOS ONE style templates can be found at

https://journals.plos.org/plosone/s/file?id=wjVg/PLOSOne_formatting_sample_main_body.pdf and

https://journals.plos.org/plosone/s/file?id=ba62/PLOSOne_formatting_sample_title_authors_affiliations.pdf

2. In your manuscript, please provide additional information regarding the specimens used in your study. Ensure that you have reported human remain specimen numbers and complete repository information, including museum name and geographic location.

If permits were required, please ensure that you have provided details for all permits that were obtained, including the full name of the issuing authority, and add the following statement:

'All necessary permits were obtained for the described study, which complied with all relevant regulations.'

If no permits were required, please include the following statement:

'No permits were required for the described study, which complied with all relevant regulations.'

For more information on PLOS One's requirements for paleontology and archeology research, see https://journals.plos.org/plosone/s/submission-guidelines#loc-paleontology-and-archaeology-research.

3. Thank you for stating the following financial disclosure:

“This study was carried out within the framework of the Mohács 500 project, with the support of the Ministry of Agriculture of the Hungarian Government (government decree nos. 1159/2023., IV. 27. and 1288/2024., IX. 19.), awarded to GP. This work was also funded by the University Research Scholarship Programme – Cooperative Doctoral Programme 2024 (2024-2.1.2-EKÖP-KDP) of the Hungarian Ministry of Culture and Innovation (grant agreement no. EKÖP-KDP-24-SZTE-2) to MAM, by the University of Szeged Open Access Fund (grant agreement no. 8439) to MAM, and by the STARTING_25 sub-programme of the National Research Excellence Programme, under the management of the Hungarian National Research, Development and Innovation Office (grant agreement no. STARTING 153194) to OS. The funders had no role in the study design; in the collection, analysis, and interpretation of data; in the writing of the manuscript; and in the decision to submit the manuscript for publication.”

We note that one or more of the authors is affiliated with the funding organization, indicating the funder may have had some role in the design, data collection, analysis or preparation of your manuscript for publication; in other words, the funder played an indirect role through the participation of the co-authors. If the funding organization did not play a role in the study design, data collection and analysis, decision to publish, or preparation of the manuscript and only provided financial support in the form of authors' salaries and/or research materials, please do the following:

1. Review your statements relating to the author contributions, and ensure you have specifically and accurately indicated the role(s) that these authors had in your study. These amendments should be made in the online form.

2. Confirm in your cover letter that you agree with the following statement, and we will change the online submission form on your behalf:

“The funder provided support in the form of salaries for authors [insert relevant initials], but did not have any additional role in the study design, data collection and analysis, decision to publish, or preparation of the manuscript. The specific roles of these authors are articulated in the ‘author contributions’ section.

4. We note that Figures 1 and 3 and supplementary figures 1 - 8 in your submission contain copyrighted images. All PLOS content is published under the Creative Commons Attribution License (CC BY 4.0), which means that the manuscript, images, and Supporting Information files will be freely available online, and any third party is permitted to access, download, copy, distribute, and use these materials in any way, even commercially, with proper attribution. For more information, see our copyright guidelines: http://journals.plos.org/plosone/s/licenses-and-copyright.

We require you to either (1) present written permission from the copyright holder to publish these figures specifically under the CC BY 4.0 license, or (2) remove the figures from your submission:

1. You may seek permission from the original copyright holder of Figure(s) [#] to publish the content specifically under the CC BY 4.0 license.

We recommend that you contact the original copyright holder with the Content Permission Form (http://journals.plos.org/plosone/s/file?id=7c09/content-permission-form.pdf) and the following text:

“I request permission for the open-access journal PLOS ONE to publish XXX under the Creative Commons Attribution License (CCAL) CC BY 4.0 (http://creativecommons.org/licenses/by/4.0/). Please be aware that this license allows unrestricted use and distribution, even commercially, by third parties. Please reply and provide explicit written permission to publish XXX under a CC BY license and complete the attached form.”

Please upload the completed Content Permission Form or other proof of granted permissions as an "Other" file with your submission.

In the figure caption of the copyrighted figure, please include the following text: “Reprinted from [ref] under a CC BY license, with permission from [name of publisher], original copyright [original copyright year].”

2. If you are unable to obtain permission from the original copyright holder to publish these figures under the CC BY 4.0 license or if the copyright holder’s requirements are incompatible with the CC BY 4.0 license, please either i) remove the figure or ii) supply a replacement figure that complies with the CC BY 4.0 license. Please check copyright information on all replacement figures and update the figure caption with source information. If applicable, please specify in the figure caption text when a figure is similar but not identical to the original image and is therefore for illustrative purposes only.

5. We note that Figure 1 in your submission contain map images which may be copyrighted. All PLOS content is published under the Creative Commons Attribution License (CC BY 4.0), which means that the manuscript, images, and Supporting Information files will be freely available online, and any third party is permitted to access, download, copy, distribute, and use these materials in any way, even commercially, with proper attribution. For these reasons, we cannot publish previously copyrighted maps or satellite images created using proprietary data, such as Google software (Google Maps, Street View, and Earth). For more information, see our copyright guidelines: http://journals.plos.org/plosone/s/licenses-and-copyright.

We require you to either (1) present written permission from the copyright holder to publish these figures specifically under the CC BY 4.0 license, or (2) remove the figures from your submission:

1. You may seek permission from the original copyright holder of Figure(s) [#] to publish the content specifically under the CC BY 4.0 license.

We recommend that you contact the original copyright holder with the Content Permission Form (http://journals.plos.org/plosone/s/file?id=7c09/content-permission-form.pdf) and the following text:

“I request permission for the open-access journal PLOS ONE to publish XXX under the Creative Commons Attribution License (CCAL) CC BY 4.0 (http://creativecommons.org/licenses/by/4.0/). Please be aware that this license allows unrestricted use and distribution, even commercially, by third parties. Please reply and provide explicit written permission to publish XXX under a CC BY license and complete the attached form.”

Please upload the completed Content Permission Form or other proof of granted permissions as an "Other" file with your submission.

In the figure caption of the copyrighted figure, please include the following text: “Reprinted from [ref] under a CC BY license, with permission from [name of publisher], original copyright [original copyright year].”

2. If you are unable to obtain permission from the original copyright holder to publish these figures under the CC BY 4.0 license or if the copyright holder’s requirements are incompatible with the CC BY 4.0 license, please either i) remove the figure or ii) supply a replacement figure that complies with the CC BY 4.0 license. Please check copyright information on all replacement figures and update the figure caption with source information. If applicable, please specify in the figure caption text when a figure is similar but not identical to the original image and is therefore for illustrative purposes only.

The following resources for replacing copyrighted map figures may be helpful:

USGS National Map Viewer (public domain): http://viewer.nationalmap.gov/viewer/

The Gateway to Astronaut Photography of Earth (public domain): http://eol.jsc.nasa.gov/sseop/clickmap/

Maps at the CIA (public domain): https://www.cia.gov/library/publications/the-world-factbook/index.html and https://www.cia.gov/library/publications/cia-maps-publications/index.html

NASA Earth Observatory (public domain): http://earthobservatory.nasa.gov/

Landsat: http://landsat.visibleearth.nasa.gov/

USGS EROS (Earth Resources Observatory and Science (EROS) Center) (public domain): http://eros.usgs.gov/#

Natural Earth (public domain): http://www.naturalearthdata.com/

6. Please include a copy of Table 1 which you refer to in your text on page 14.

7. We note you have included a table to which you do not refer in the text of your manuscript. Please ensure that you refer to Table 5 in your text; if accepted, production will need this reference to link the reader to the Table.

8. If the reviewer comments include a recommendation to cite specific previously published works, please review and evaluate these publications to determine whether they are relevant and should be cited. There is no requirement to cite these works unless the editor has indicated otherwise.

9. Please review your reference list to ensure that it is complete and correct. If you have cited papers that have been retracted, please include the rationale for doing so in the manuscript text, or remove these references and replace them with relevant current references. Any changes to the reference list should be mentioned in the rebuttal letter that accompanies your revised manuscript. If you need to cite a retracted article, indicate the article’s retracted status in the References list and also include a citation and full reference for the retraction notice.

Additional Editor Comments:

This is an excellent paper that substantially adds to our efforts in the recovery and analysis of human remains from both, forensic and archaeological contetxs. The authors provide a new, improved methodology on how to successfully recover and study such contexts. I agree with both reviewers that this is a very important piece without major flaws. However, as R2 indicated the authors are trying maybe a bit too much to make this methodology universal and applicable to all possible cases which might not always be the case. Additionaly, this method also requires a certain technical background that might not be available to all those dealing with such burials. Therefore, I would suggest that the authors maybe 'tone' down a bit certain parts of the text. There are also some other minor suggestions and comments by both reviewers that I think would improve the manuscript in certain points.

[Note: HTML markup is below. Please do not edit.]

Reviewers' comments:

Reviewer's Responses to Questions

**Comments to the Author**

1. Is the manuscript technically sound, and do the data support the conclusions?

The manuscript must describe a technically sound piece of scientific research with data that supports the conclusions. Experiments must have been conducted rigorously, with appropriate controls, replication, and sample sizes. The conclusions must be drawn appropriately based on the data presented.

Reviewer #1: Yes

Reviewer #2: No

**********

2. Has the statistical analysis been performed appropriately and rigorously?

Reviewer #1: Yes

Reviewer #2: I Don't Know

**********

3. Have the authors made all data underlying the findings in their manuscript fully available?

The PLOS Data policy requires authors to make all data underlying the findings described in their manuscript fully available without restriction, with rare exception (please refer to the Data Availability Statement in the manuscript PDF file). The data should be provided as part of the manuscript or its supporting information, or deposited to a public repository. For example, in addition to summary statistics, the data points behind means, medians and variance measures should be available. If there are restrictions on publicly sharing data—e.g. participant privacy or use of data from a third party—those must be specified.

Reviewer #1: Yes

Reviewer #2: Yes

**********

4. Is the manuscript presented in an intelligible fashion and written in standard English?

PLOS ONE does not copyedit accepted manuscripts, so the language in submitted articles must be clear, correct, and unambiguous. Any typographical or grammatical errors should be corrected at revision, so please note any specific errors here.

Reviewer #1: Yes

Reviewer #2: Yes

**********

5. Review Comments to the Author

Please use the space provided to explain your answers to the questions above. You may also include additional comments for the author, including concerns about dual publication, research ethics, or publication ethics. (Please upload your review as an attachment if it exceeds 20,000 characters)

Reviewer #1: Thank you for this useful paper. Please, see my few comments and suggestions in attached document. I only have some minor comments on rephrasing some "unfortunate" wording and a few comments on technical procedure, all of them rather minor changes.

Reviewer #2: In general, this article gives a great method for reassociation with commingled remains. I am a firm believer that there are multiple ways to approach commingled remains, and that there is no single solution to how to approach them in terms of analysis or interpretation. The method described within provides another tool in the skillset that may be utilized by practitioners, and therefore is worthy of publication.

I agree that there is a lack of consistent terminology used for commingled remains, and that this is a result of different training and the relative youth of the study of commingled remains as a focus. Until around the 1990s, commingled remains were typically relegated to appendices or stored without significant study since the assemblages were thought to be too difficult or not to produce significant results. It is bound to be the case that consistent methodology would not be forthcoming. They’re probably also correct that the time is ripe for developing terminological consistency, much as Buikstra and Ubelaker did in 1994 for general bioarchaeological recording. I’m not sure that an article like this is the place to provide that, since they cannot approach all situations that produce commingled remains. I think that type of volume will be coming in the next few years though, and work such as this article will help to formulate those guidelines.

I feel that the authors are very committed to their method and see it as the best approach to resolve commingling and assist in reassociation. It looks great, but it isn’t a universal fix and the authors should acknowledge that there are different approaches that may be employed to good results. This method relies on a significant amount of technological expertise, including using QGIS. QGIS may not be available to all researchers and/or all researchers may not have the technological know-how to utilize this method. This is the primary issue that I have with this article, otherwise, the method seems sound and the supplementary text is well done and provides a step-by-step process to employ the authors’ methodology.

The case study used here has a good number of articulated elements. I think if they frame their work such that it is good for commingled remains where there is significant articulation, it would be good. Fragmentation complicates this picture immensely. I don’t suggest that the authors attempt to apply their methodology to cases of fragmented and commingled remains, but since fragmentation also generally accompanies commingling, they should specify which types of assemblages their method would be best applied to. If they choose to address this, they should identify how such methodology would be utilized on extreme processing assemblages from the American Southwest, for example (e.g., Kuckelman, Lightfoot, and Martin 2000; Martin, Akins, and Toll 2014; Martin and Osterholtz 2020).

I like the numbering and identification system that the authors propose, but I think this is more effective for a commingled situation with more complete elements.

Generally, I like this article quite a bit. It’s well founded and provides a great case study. I think the authors should reframe it a bit to indicate that it is a method that works well with a particular type of commingled assemblage. Once that is done, I think this will be a great contribution to the wide-ranging body of literature that has come out in the past 20 years on commingling.

One general note:

MNI is determined, not estimated. Since MNI is a measure of the observed number of elements (or features/zones), it is determined. MLNI is estimated, though. I’d caution the authors to be careful in their language.

Buikstra, Jane, and D H. Ubelaker. 1994. Standards for Data Collection from Human Skeletal Remains. (Fayetteville).

Kuckelman, Kristin A., Ricky R. Lightfoot, and Debra L. Martin. 2000. "Changing Patterns of Violence in the Northern San Juan Region." Kiva 66 (1): 147–165.

Martin, Debra L., Nancy J. Akins, and H. Wolcott Toll. 2014. "Disarticulated and disturbed, processed and eaten? Cautionary notes from the La Plata assemblage (AD 1000–1150)." In Commingled and disarticulated human remains, edited by Anna J. Osterholtz, Kathryn M. Baustian and Debra L. Martin, 129–147. New York: Springer.

Martin, Debra L., and Anna J. Osterholtz. 2020. "The Poetics of Corpse Fragmentation and Processing in the Ancient Southwest." In The Poetics of Processing: Memory Formation, Cosmology, and the Handling of the Dead., edited by Anna J. Osterholtz, 85–102. Boulder, CO: University Press of Colorado.

**********

6. PLOS authors have the option to publish the peer review history of their article (what does this mean?). If published, this will include your full peer review and any attached files.

If you choose “no”, your identity will remain anonymous but your review may still be made public.

**Do you want your identity to be public for this peer review?**  For information about this choice, including consent withdrawal, please see our Privacy Policy.

Reviewer #1: No

Reviewer #2: No

**********

[NOTE: If reviewer comments were submitted as an attachment file, they will be attached to this email and accessible via the submission site. Please log into your account, locate the manuscript record, and check for the action link "View Attachments". If this link does not appear, there are no attachment files.]

To ensure your figures meet our technical requirements, please review our figure guidelines: https://journals.plos.org/plosone/s/figures

You may also use PLOS’s free figure tool, NAAS, to help you prepare publication quality figures: https://journals.plos.org/plosone/s/figures#loc-tools-for-figure-preparation.

NAAS will assess whether your figures meet our technical requirements by comparing each figure against our figure specifications.

Attachment Submitted filename: Plos One Review De Andres et al.docx

# Author comment: Author response to Decision Letter 1

**DOI:** 10.1371/journal.pone.0353420.r002

5 Jun 2026

Dr. Mario Novak, PhD

Academic Editor

PLOS ONE

Dear Dr. Novak,

We are very grateful for your and the reviewers’ insightful and constructive comments regarding our manuscript entitled “New Integrative Methodology for the excavation and study of mass graves and partially commingled remains” that was submitted to PLOS ONE (manuscript ID: PONE-D-26-10119). The main text has been modified following the suggestions. The revised files have been uploaded to the submission site of the journal. In addition, Journal Requirement number 3 demanded a modification on the financial disclosure, we modified it as requested, however we would like to highlight that the new statement is only partially accurate as the funding mentioned in the original form of our financial disclosure only refers to an Open Access fund, granted by the University of Szeged to the corresponding author. The funder provided support in the form of salaries for several authors [MAM, LK, OS, VV, RK, BT and GP], but did not have any additional role in the study design, data collection and analysis, decision to publish, or preparation of the manuscript. We are sure that you and the reviewers helped us to improve the quality of our manuscript.

Response to the Academic Editor’s comments

1) This is an excellent paper that substantially adds to our efforts in the recovery and analysis of human remains from both, forensic and archaeological contetxs. The authors provide a new, improved methodology on how to successfully recover and study such contexts. I agree with both reviewers that this is a very important piece without major flaws. However, as R2 indicated the authors are trying maybe a bit too much to make this methodology universal and applicable to all possible cases which might not always be the case.

We would like to thank the Academic Editor for the positive evaluation and constructive comments on our work. We agree that this method is not universal, and we would like to emphasize that it was never our intention to present it in this manner. We are fully aware of its limitations and the fact that it can only be applied successfully in cases meeting certain criteria. This was indicated in the Discussion section (lines 527–536):

“This method can be applied across a wide range of contexts, including primary unaltered mass graves/multiple burials and those exhibiting varying degrees of commingling. However, factors such as bone preservation and the high degree of commingling may influence its applicability and effectiveness. In particular, poor preservation – where bone identification and assessments of anatomical compatibility are hindered – may substantially reduce the analytical potential. Importantly, the tools presented here have not yet been tested on completely commingled assemblages in which no anatomical connections are recorded in the field, and only limited utility should be expected in such cases. In addition to commingled human skeletal remains, the applicability of this method in cases involving zooarchaeological, paleontological, or paleoanthropological remains is yet to be tested.”

We consider the application of GIS technology to be a substantial aid in the reassociation process as it can compensate for the lost efficacy of other techniques– such as osteometry or visual comparisons of articulating or paired bones– when dealing with large assemblages.

However, we do not support the idea that GIS technology should replace existing tools such as osteometric sorting or visual comparison of articulating elements and paired bones. On the contrary, we support their combined use, as stated in the Discussion section (lines 499–505):

“Despite its advantages, the methodology has inherent limitations. In agreement with previous scholars [49,50], we consider it as part of a broader, integrative protocol in which additional analytical tools should be employed to achieve more comprehensive documentation and stronger evidentiary support for reassociation [36,65]. During laboratory analyses, the proposed approach can be effectively combined with established methods such as osteometric sorting, articulation, visual pair-matching, process of elimination, and, where feasible, DNA analysis, thereby forming an efficient and robust workflow (see Fig 8).”

Our proposal is therefore based on integrating the described techniques and methods into a broader and structured workflow intended to facilitate the work of specialists facing similar situations. We attempted to summarize this workflow in Fig. 9:

“Algorithm for the reassociation of commingled human skeletal remains. In this step-by-step workflow, both DNA and process of elimination are ommitted as they are to be used separately. For secondary burial or ossariums, osteometry should be the first step, as spatial relationships for two elements cannot guarantee belonging to a same individual. The visual assessment when both options are available starts with articulating elements as this is a less subjective technique and continues with comparison between pairs. If the result of both examinations is satisfactory, a proposed match can be considered a successful reassociation.”

To make these ideas clearer, we propose the following changes in the revised version of the manuscript:

In the discussion section, lines 454–456 of the revised version of our manuscript, we have inserted the following sentence:

Original version (lines 453–454):

“The methodology presented here is suitable for both small and large commingled assemblages.”

Revised version (lines 454–456):

“The methodology presented here is suitable for both small and large commingled assemblages, increasing the efficiency of reassociations when combined with other techniques”

2) Additionaly, this method also requires a certain technical background that might not be available to all those dealing with such burials.

Regarding the technical skills required to apply this methodology effectively, we agree with both the Editor and Reviewer#2 that a certain level of technical knowledge is necessary to navigate the software and conduct the proposed analyses. However, precisely because only a basic level of GIS proficiency is required, we consider this to be an advantage compared to other approaches that also incorporate GIS technology (e.g., Tuller&Hofmeister, 2014; Voeller, 2024). To make the methodology accessible even to readers without prior experience using GIS software, we included a Supplementary File covering the most fundamental operations required to record and manage data using georeferenced photographs of the site, which are already common practice in archaeological documentation.

In agreement with the comment from Reviewer#2, we modified lines 462–471 in the revised version of the manuscript to emphasize that a certain level of knowledge is indeed required:

Original text (460–467):

“As described earlier, our methodology incorporates the use of GIS software – a tool that has been applied to commingled assemblages and mass grave investigations before [49,50]; however, such approaches often require advanced proficiency in GIS and, in some cases, a strong statistical background. In contrast, our recommendations allow any specialist with basic GIS skills (e.g., creating polygon layers to highlight the different units and recording related attribute data) to perform the necessary tasks (see Supplementary File 1). Although a more detailed understanding of software functions and statistical principles is advantageous and can facilitate reassociation, neither is indispensable for applying the methodology proposed here.”

Revised text (lines 462–471):

“As described earlier, our methodology incorporates the use of GIS software – a tool that has been applied to commingled assemblages and mass grave investigations before [49,50]; however, such approaches often require advanced proficiency in GIS and, in some cases, a strong statistical background. In contrast, our recommendations require only a basic knowledge of GIS technology and elementary skills on handling this type of software (e.g., creating polygon layers to highlight the different units and recording related attribute data) to perform the necessary tasks (see Supplementary File 1). We recommend QGIS as it is a freely available software that can be installed in most devices. Although a more detailed understanding of software functions and statistical principles is advantageous and can facilitate reassociation, neither is indispensable for applying the methodology proposed here.”

References:

Tuller H, Hofmeister U. Spatial Analysis of Mass Grave Mapping Data to Assist in the Reassociation of Disarticulated and Commingled Human Remains. In Adams BJ, Byrd J, editors. Commingled Human Remains.: Academic Press; 2014. p. 7-32.

Voeller S. The use of GIS for cases of comingling. In Ross AH, Byrd JH, editors. Methodological and Technological Advances in Death Investigations.: Academic Press; 2024. p. 225-246.

3) Therefore, I would suggest that the authors maybe 'tone' down a bit certain parts of the text. There are also some other minor suggestions and comments by both reviewers that I think would improve the manuscript in certain points.

We appreciate this suggestion and have revised the relevant sections of the manuscript to clarify our position and avoid overstating the universality of the proposed methodology. In addition, we have incorporated the remaining comments and corrections suggested by both reviewers throughout the revised version of the manuscript. Furthermore, we corrected two words that were wrongly spelled or misused (lines 423 and 527), these are indicated with a comment reading “Typo corrected”. Please find our detailed response to the Reviewers’ comments below.

Response to the Reviewers’ comments

Reviewer #1:

1. In following, there are some suggestions of changes and rephrasing in the in general well written and interesting paper. None of suggested is vital, overall recommendation is “minor revisions”

We would like to thank Reviewer for the positive evaluation of our manuscript, as well as for the constructive comments and suggestions that helped us improve the clarity and quality of the paper.

2. Abstract: rephrase the “reliable results”. I deeply hope, there are no analyses aiming to provide any other, but reliable results. Sound weird.

We thank the Reviewer for this comment and agree that, in principle, analyses should aim to provide reliable results. We have therefore removed the word “reliable” from line 27 in the revised version of the manuscript.

3. Abstract: rephrase “recovered”. There is NO WAY to increase the number of recovered skeletons. You only can increase the number of identified ones…

We thank the Reviewer for this comment too and fully agree with it. We have replaced the word “recovered” with “individualized” in line 35 of the revised version of our manuscript.

Main text:

4. Lines 100-108: please rephrase, this is surely true, but NOT only for plural burials, even if there it makes the huge difference.

We appreciate the indication and agree that the variables listed in the original text may affect the excavation of any burial context, although they are particularly relevant in plural burials. We therefore rephrased the text as follows:

Original text:

“Distinct variables can potentially complicate the excavation and exhumation of remains interred in plural burials [31].”

Revised text:

“Distinct variables can potentially complicate the excavation and exhumation of remains interred in any burial context, especially those containing multiple individuals [31].”

5. Lines 246-253: ethics is nowadays doubtlessly a relevant topic, even for human remains of >500 years of age, however, it is not a part of the main publication body. Unless Plos has a new way of treating it? Please, move to the end or to the beginning, outside the main text body.

We thank the Reviewer for this comment. However, this section was added following a request from the editorial office.

6. Line 291: rather 00001 to 99999, isn’t it? Otherwise you’re going to have a problem with any programmes from Microsoft.

We thank the Reviewer for their comment on this matter. Since we mainly worked within QGIS, this issue did not arise during our analyses. However, we agree that if the dataset is exported to other software (e.g., Microsoft Excel), the absence of leading zeros may indeed create complications. Therefore, we have included this suggestion into line 291 of the revised manusctipt.

Fig 3.:

7. Bone type in full names? LHu/RHu for right and left humerus would do and, on your thousands of lines, save a lot of data

We are grateful for this suggestion and agree that reducing the number of characters inserted during data recording can improve efficiency. However, throughout our project we consistently used full names, and the figures presented in the manuscript were generated directly from our original QGIS dataset. To preserve consistency with the original workflow, we retained the full names in the examples presented.

Additionally, one advantage of the proposed system is the relatively short length of the coding lines used within the QGIS filters, as represented in Figure 6. Consequently, the difference in character length between full names and abbreviations would not substantially affect the complexity of the coding process. Nevertheless, we agree that abbreviations are entirely feasible, and therefore added the following clarification to the subsection “Bone type and laterality” in Table 1: “This can be the bone’s full name or any unique abbreviation to each element.”

8. Possible connection: “=” implies, there is FOR SURE a connection, being “the same”. We cannot say this FOR SURE by any osteology alone, without DNA, this is always merely “99.9%”. Rather some marking such as “≈” or “→”

We thank the Reviewer for this observation and agree that a different symbol would better represent a proposed rather than definitive association. Accordingly, we replaced the “=” symbol with “→” in the subsection “Possible connection with other elements” of Table 1.

9. Miscellaneous: is it reasonable to mix up information directly relevant to identification of individuals (such as age-at-death, sex, systemic pathologies) with interesting but not relevant for sorting information (such as cut marks or osteomyelitis)? Then, this information misses their main purpose, to identify the missing parts, and go on to another topic, the investigation of the individuals themselves. This is not your goal here, otherwise, the system would need a lot of other info, too…

We truly appreciate this insightful comment. Although recording such information may initially appear impractical due to the additional time and space required, our experience with Mass Grave No.3 at the Mohács National Memorial Site demonstrated that characteristics such as biological sex, age-at-death, pathological changes, or perimortem trauma can indeed play a significant role in reassociation.

In our assemblage, the majority of individuals were identified as adult males. Consequently, remains belonging to females or subadults became highly distinctive and therefore particularly useful for segregation purposes. The same logic applies to pathological changes and perimortem trauma: although some pathological changes are relatively common, severe lesions are comparatively rare, while cut marks –especially in a context where decapitation represents one of the working hypotheses– may also serve as potentially segregating features.

The purpose of the “Miscellaneous” category was therefore to create a flexible field that can accommodate characteristics considered especially informative within a given assemblage, without requiring the systematic recording of all non-diagnostic features. In our case, for example, only females and subadults were specifically noted, whereas the category was intentionally left blank for adult males by default. We believe this approach allows future users to adapt the system according to the specific characteristics and research priorities of their own assemblages.

10. Line 303ff: I miss any information on hands and feet. A difficult topic, not really possible to solve to 100% in a mass gr

Attachment Submitted filename: Response to Reviewers.docx

# Peer review: Decision Letter 1

**DOI:** 10.1371/journal.pone.0353420.r003

**Authors:** Mario Novak, Mario Novak

24 Jun 2026

New Integrative Methodology for the excavation and study of mass graves and partially commingled remains

PONE-D-26-10119R1

Dear Dr. De  Andres Montero,

We’re pleased to inform you that your manuscript has been judged scientifically suitable for publication and will be formally accepted for publication once it meets all outstanding technical requirements.

Within one week, you’ll receive an e-mail detailing the required amendments. When these have been addressed, you’ll receive a formal acceptance letter and your manuscript will be scheduled for publication.

An invoice will be generated when your article is formally accepted. Please note, if your institution has a publishing partnership with PLOS and your article meets the relevant criteria, all or part of your publication costs will be covered. Please make sure your user information is up-to-date by logging into Editorial Manager at Editorial Manager® and clicking the ‘Update My Information' link at the top of the page. For questions related to billing, please contact billing support.

If your institution or institutions have a press office, please notify them about your upcoming paper to help maximize its impact. If they’ll be preparing press materials, please inform our press team as soon as possible -- no later than 48 hours after receiving the formal acceptance. Your manuscript will remain under strict press embargo until 2 pm Eastern Time on the date of publication. For more information, please contact onepress@plos.org.

Kind regards,

Mario Novak

Academic Editor

PLOS One

Additional Editor Comments (optional):

Reviewers' comments:

Reviewer's Responses to Questions

**Comments to the Author**

1. If the authors have adequately addressed your comments raised in a previous round of review and you feel that this manuscript is now acceptable for publication, you may indicate that here to bypass the “Comments to the Author” section, enter your conflict of interest statement in the “Confidential to Editor” section, and submit your "Accept" recommendation.

Reviewer #1: All comments have been addressed

Reviewer #2: All comments have been addressed

**********

2. Is the manuscript technically sound, and do the data support the conclusions?

The manuscript must describe a technically sound piece of scientific research with data that supports the conclusions. Experiments must have been conducted rigorously, with appropriate controls, replication, and sample sizes. The conclusions must be drawn appropriately based on the data presented.

Reviewer #1: Yes

Reviewer #2: Yes

**********

3. Has the statistical analysis been performed appropriately and rigorously?

Reviewer #1: Yes

Reviewer #2: I Don't Know

**********

4. Have the authors made all data underlying the findings in their manuscript fully available?

The PLOS Data policy requires authors to make all data underlying the findings described in their manuscript fully available without restriction, with rare exception (please refer to the Data Availability Statement in the manuscript PDF file). The data should be provided as part of the manuscript or its supporting information, or deposited to a public repository. For example, in addition to summary statistics, the data points behind means, medians and variance measures should be available. If there are restrictions on publicly sharing data—e.g. participant privacy or use of data from a third party—those must be specified.

Reviewer #1: Yes

Reviewer #2: Yes

**********

5. Is the manuscript presented in an intelligible fashion and written in standard English?

PLOS ONE does not copyedit accepted manuscripts, so the language in submitted articles must be clear, correct, and unambiguous. Any typographical or grammatical errors should be corrected at revision, so please note any specific errors here.

Reviewer #1: Yes

Reviewer #2: Yes

**********

6. Review Comments to the Author

Please use the space provided to explain your answers to the questions above. You may also include additional comments for the author, including concerns about dual publication, research ethics, or publication ethics. (Please upload your review as an attachment if it exceeds 20,000 characters)

Reviewer #1: (No Response)

Reviewer #2: great job in addressing comments! This is a good contribution to the literature on commingled remains and provides one more method to add to the tool kit.

**********

7. PLOS authors have the option to publish the peer review history of their article (what does this mean?). If published, this will include your full peer review and any attached files.

If you choose “no”, your identity will remain anonymous but your review may still be made public.

**Do you want your identity to be public for this peer review?**  For information about this choice, including consent withdrawal, please see our Privacy Policy.

Reviewer #1: No

Reviewer #2: No

**********

# Editor report: Acceptance letter

**DOI:** 10.1371/journal.pone.0353420.r004

**Authors:** Mario Novak, Mario Novak

PONE-D-26-10119R1

PLOS One

Dear Dr. De Andrés Montero,

I'm pleased to inform you that your manuscript has been deemed suitable for publication in PLOS One. Congratulations! Your manuscript is now being handed over to our production team.

At this stage, our production department will prepare your paper for publication. This includes ensuring the following:

* All references, tables, and figures are properly cited

* All relevant supporting information is included in the manuscript submission,

* There are no issues that prevent the paper from being properly typeset

You will receive further instructions from the production team, including instructions on how to review your proof when it is ready. Please keep in mind that we are working through a large volume of accepted articles, so please give us a few days to review your paper and let you know the next and final steps.

Lastly, if your institution or institutions have a press office, please let them know about your upcoming paper now to help maximize its impact. If they'll be preparing press materials, please inform our press team within the next 48 hours. Your manuscript will remain under strict press embargo until 2 pm Eastern Time on the date of publication. For more information, please contact onepress@plos.org.

You will receive an invoice from PLOS for your publication fee after your manuscript has reached the completed accept phase. If you receive an email requesting payment before acceptance or for any other service, this may be a phishing scheme. Learn how to identify phishing emails and protect your accounts at https://explore.plos.org/phishing.

If we can help with anything else, please email us at customercare@plos.org.

Thank you for submitting your work to PLOS ONE and supporting open access.

Kind regards,

PLOS ONE Editorial Office Staff

on behalf of

Dr. Mario Novak

Academic Editor

PLOS One
